# Chiral Chalcogen Bond Donors Based on the 4,4′-Bipyridine Scaffold

**DOI:** 10.3390/molecules24244484

**Published:** 2019-12-06

**Authors:** Robin Weiss, Emmanuel Aubert, Paola Peluso, Sergio Cossu, Patrick Pale, Victor Mamane

**Affiliations:** 1 Strasbourg Institut of Chemistry, UMR CNRS 7177, Team LASYROC, 1 rue Blaise Pascal, University of Strasbourg, 67008 Strasbourg CEDEX, France; robin.weiss@unistra.fr (R.W.); ppale@unistra.fr (P.P.); 2 Crystallography, Magnetic Resonance and Modelisations (CRM2), UMR CNRS 7036, University of Lorraine, Bd des Aiguillettes, 54506 Vandoeuvre-les-Nancy, France; emmanuel.aubert@univ-lorraine.fr; 3 Institute of Biomolecular Chemistry ICB, CNR, Secondary branch of Sassari, Traversa La Crucca 3, Regione Baldinca, 07100 Li Punti, Sassari, Italy; 4 Department of Molecular Science and Nanosystems DSMN, Ca’ Foscari University of Venice, Via Torino 155, 30172 Mestre Venice, Italy; cossu@unive.it

**Keywords:** atropisomerism, 4,4′-bipyridine, chalcogen bond, electronic circular dichroism, noncovalent interactions, organocatalysis, selenium, σ-hole

## Abstract

Organocatalysis through chalcogen bonding (ChB) is in its infancy, as its proof-of-principle was only reported in 2016. Herein, we report the design and synthesis of new chiral ChB donors, as well as the catalytic activity evaluation of the 5,5′-dibromo-2,2′-dichloro-3-((perfluorophenyl)selanyl)-4,4′-bipyridine as organocatalyst. The latter is based on the use of two electron-withdrawing groups, a pentafluorophenyl ring and a tetrahalo-4,4′-bipyridine skeleton, as substituents at the selenium center. Atropisomery of the tetrahalo-4,4′-bipyridine motif provides a chiral environment to these new ChB donors. Their synthesis was achieved through either selective lithium exchange and trapping or a site-selective copper-mediated reaction. Pure enantiomers of the 3-selanyl-4,4′-bipyridine were obtained by high performance liquid chromatography enantioseparation on specific chiral stationary phase, and their absolute configuration was assigned by comparison of the measured and calculated electronic circular dichroism spectra. The capability of the selenium compound to participate in σ-hole-based interactions in solution was studied by ^19^F NMR. Even if no asymmetric induction has been observed so far, the new selenium motif proved to be catalytically active in the reduction of 2-phenylquinoline by Hantzsch ester.

## 1. Introduction

**Figure 1:**
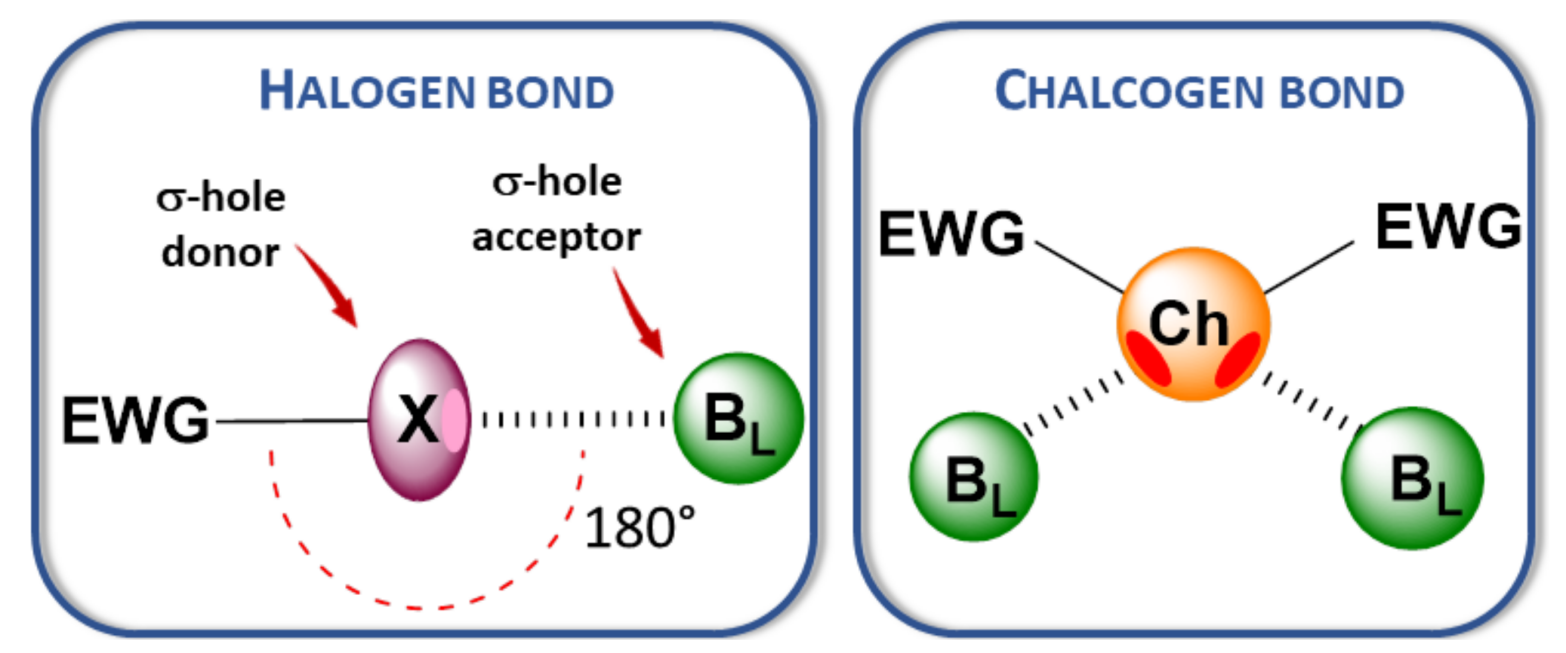
Halogen and chalcogen bonds (EWG: Electron withdrawing group, B_L_: Lewis base).

The term chalcogen bond (ChB) describes the intermolecular interaction occurring between a Lewis base (B_L_) and a group 16 atom (S, Se, Te) which behaves like Lewis acid, according to the recommendations of the International Union of Pure and Applied Chemistry (IUPAC) (*net attractive interaction between an electrophilic region associated with a chalcogen atom in a molecular entity and a nucleophilic region in another, or the same, molecular entity*) [1,2,3]. ChB belongs to the family of σ-hole-based interactions [4], among which the most prominent and most studied is halogen bonding (XB) [5,6,7]. ChB possesses many similarities with XB [8,9], but with a different interaction pattern, because up to two σ-hole···B_L_ axes can be identified on a Ch atom due to its divalent nature (Figure 1). Although noncovalent contacts involving sulfur have been recognized in biological molecules during the past decade [10], it is only recently that a few applications involving ChB in solid state [11] and in solution [12] have appeared, in particular those involving heavier elements such as selenium and tellurium.

Among the applications based on σ-hole interactions in solution, catalysis represents an active research area. While several catalysts acting as XB donor have been described during the last decade [6,13,14], the first examples of ChB-based catalysis have been very recently reported by the groups of Matile [15,16,17,18], Huber [19,20,21], and Wang [22]. Other recent applications of ChB concern anion binding [23,24] and transport [25,26,27].

Introduction of chirality in systems able to interact through noncovalent bonds can have a significant impact in many areas, including biology, molecular recognition, catalysis, and material sciences [28,29]. Unlike XB-based processes, for which a number of chiral donor molecules were developed in the last few years [5,6,12,30], only very few applications using chiral ChB donors have been reported. Beer’s group described a chiral selenium-containing receptor for enantiomeric discrimination of dicarboxylates [24]. Following previous studies on XB-based high performance liquid chromatography (HPLC) enantioseparations [31,32,33,34,35,36], our groups demonstrated that ChB can participate in the recognition process underlying the enantioseparation of sulfur-containing atropisomeric 4,4′-bipyridine **1** on a cellulose-based chiral stationary phase [37].

**Figure 2:**
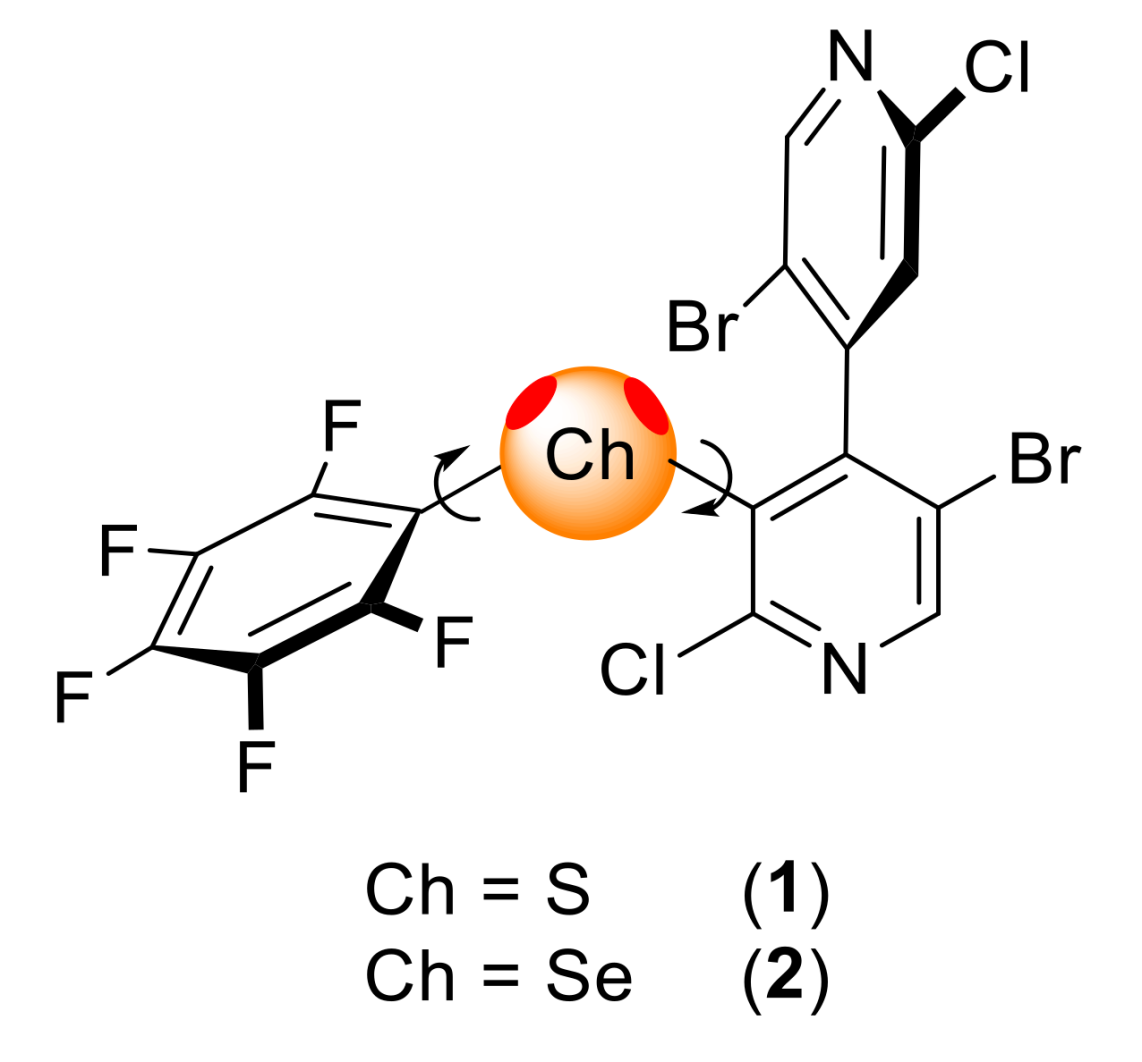
Chiral 3-chalcogeno-4,4′-bipyridines considered in this work.

On this basis, we report here our results on the synthesis of thio- and seleno-substituted 4,4′-bipyridines as ChB donors and the evaluation of compounds **1** and **2** as organocatalysts in the reduction of 2-phenylquinoline (Figure 2). For this purpose, an improved synthetic protocol for the synthesis of compound **1** was developed and applied to the preparation of derivative **2**. Compounds **1** and **2** were active as ChB donor catalysts in the reduction of 2-phenylquinoline, but without enantioselectivity induction. Moreover, studying the behavior of **1** and **2** in the presence of chloride ions by ^19^F NMR revealed that these compounds form σ-hole bonds in solution.

## 2. Results

### 2.1. Synthesis

**Scheme 1:**
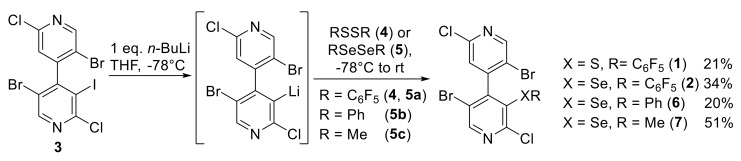
Synthesis of bipyridine derivatives **1**, **2**, **6**, and **7** through a lithiated intermediate.

The thio-substituted 4,4′-bipyridine **1** was prepared in 21% yield from the readily available pentahalogeno-4,4′-bipyridine **3** [38] by selective iodine/lithium exchange and trapping with bis(pentafluorophenyl)disulfide **4**, according to our own method [37]. The same conditions were used to prepare the selenium derivatives **2**, **6**, and **7** starting from **3** and the corresponding diselenides **5a**–**c** (Scheme 1). Although the expected products could be observed by NMR in the reaction mixtures, their purification proved to be very challenging, and the final products could only be isolated with, respectively, 34, 20, and 51% yield through HPLC purification.

These difficulties and the resulting modest yields led us to consider the use of a cross-coupling reaction involving 4,4′-bipyridine **3** as starting material. Indeed, we already succeeded in selective Pd-catalyzed couplings on halogenated 4,4′-bipyridines [38,39]. However, the introduction of SC_6_F_5_ and SeC_6_F_5_ groups onto haloaromatics through a cross-coupling reaction is poorly described in the literature. Yagupolskii and coworkers described their copper-mediated coupling with several iodoaromatics [40], and later, Haupt and coworkers applied this methodology to the polyfunctionalization of corannulene [41]. On these bases, we screened several conditions starting from **3** (Table 1), focusing on selectivity, which obviously is a major issue considering the number of potentially reactive carbon–halogen positions.

Methodology optimization was performed by using disulfide **4** (entries 1–10) in order to find the best conditions for the selective mono-functionalization. The reactive copper complex [CuSC_6_F_5_] was in situ produced by mixing **4** with two equivalents of copper as a powder in N-methyl-2-pyrrolidone (NMP) at room temperature for 3 h. Bipyridine **3** was then added and the mixture was heated for 16 h at a given temperature. We first evaluated the effect of the temperature on the reactivity and selectivity starting with 1.3 eq. of **4** (entries 1–3). At 80 °C, the reaction was not complete and a substantial amount of starting product was still present (entry 1). At 100 °C, the conversion was higher but the over-coupling compound **8** appeared due to the insertion of a second SC_6_F_5_ group on the more substituted and electron-poor pyridine ring (see Supplementary Information for NMR details). Under these conditions, the expected thiobipyridine **1** could, nevertheless, be isolated in good yield (74%, entry 2). Increasing further the temperature (130 °C) induced more formation of **8**, allowing its isolation with 39% yield (entry 3). While keeping the temperature at 100 °C, the amount of **4** was increased from 0.5 to 4.0 eq. (entries 4–8) in order to evaluate the effect of electrophile concentration. With half equivalent of **4** (i.e., 1 eq. of the active copper species), the conversion was very low (entry 4). Slight improvement was obtained by increasing the amount of **4** to 0.8 eq. (entry 5) and then to 1.0 eq. (entry 6). With 2.0 and 4.0 eq. (entries 7 and 8), the starting bipyridine **3** was completely consumed, but the amount of **8** increased with the increasing amount of copper reagent. No improvement was observed by increasing the temperature to 130 °C while reducing the amount of **4** to 0.5 equiv (entry 9). Rewardingly, with 2.0 eq., the crude reaction mixture was clean and **1** could thus be isolated with 67% yield (entry 7). Mixing all the components without preformation of [CuSC6F5] led to a higher formation of **8** (entry 10). Under conditions optimized for mono-coupling (as in entry 2), the selenobipyridine **2** was obtained with 81% yield, starting from diselenide **5a** (entry 11). The bis-coupling product of selenium, analog to **8**, was not observed at 100 °C and could not be obtained at 130 °C because decomposition mostly occurred.

### 2.2. Analyses

#### 2.2.1. HPLC Enantioseparation and Electronic Circular Dichroism (ECD)

The atropisomeric pairs of bipyridines **1**, **2**, **6**, and **7** were obtained in good amounts by means of HPLC multimilligram enantioseparation under normal phase elution conditions. As we previously reported [37], the enantiomers of compound **1** (>99% ee) were recovered by using Chiralpak IA (immobilized amylose tris(3,5-dimethylphenylcarbamate)) as chiral column. On the contrary, the enantioseparations of compounds **2**, **6**, and **7** were performed on Chiralpak IC (immobilized cellulose tris(3,5-dichlorophenylcarbamate)) (Table 2).

**Figure 3:**
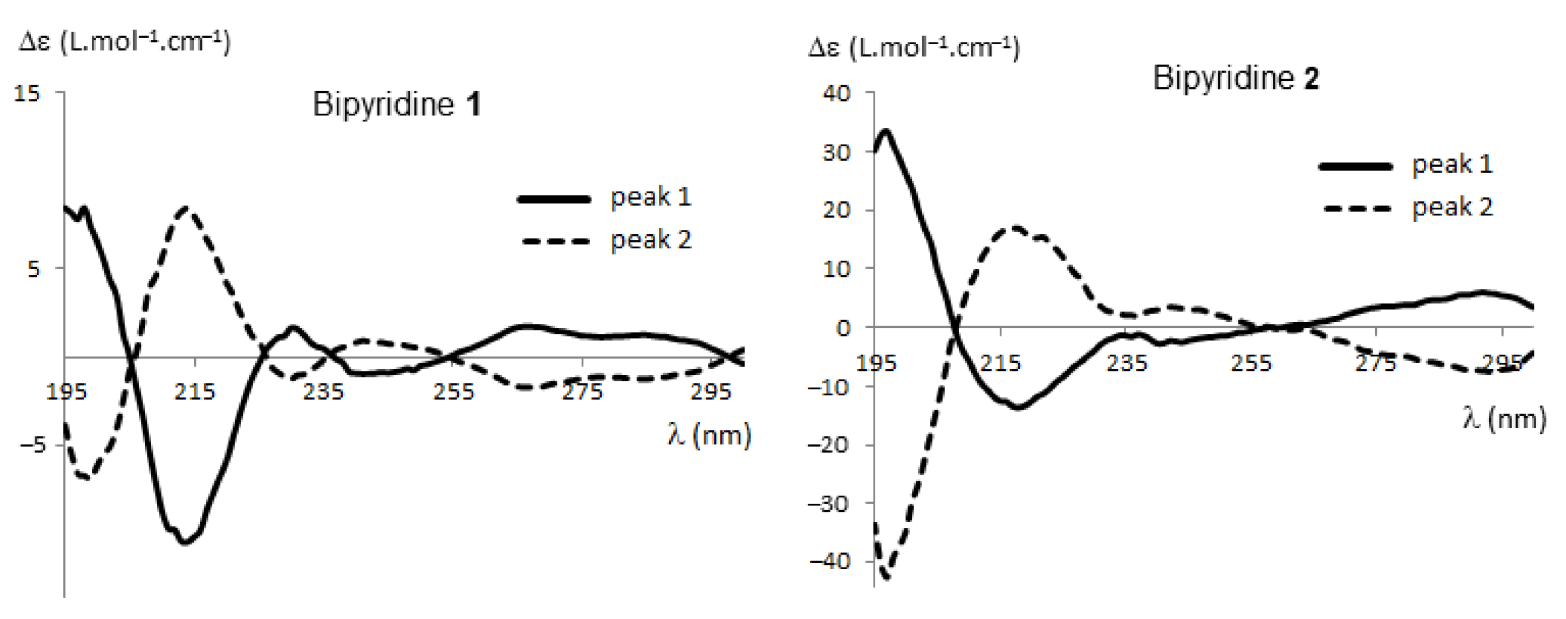
Electronic circular dichroism (ECD) spectra of the enantiomers of bipyridines **1** and **2**.

As we demonstrated, the absolute configurations of HPLC-separated enantiomers of polyhalogenated 4,4′-bipyridines can be successfully determined by electronic circular dichroism (ECD) coupled with time-dependent density functional theory (TD-DFT) calculations [37,38,39,42]. These techniques were therefore applied to each enantiomer of **1**, **2**, **6**, and **7.** Recorded in ethanol, their ECD spectra showed the expected opposite traces for the two atropisomers (Figure 3; for ECD spectra of **6** and **7**, see Figures S1 and S2 in Supplementary Information).

**Figure 4:**
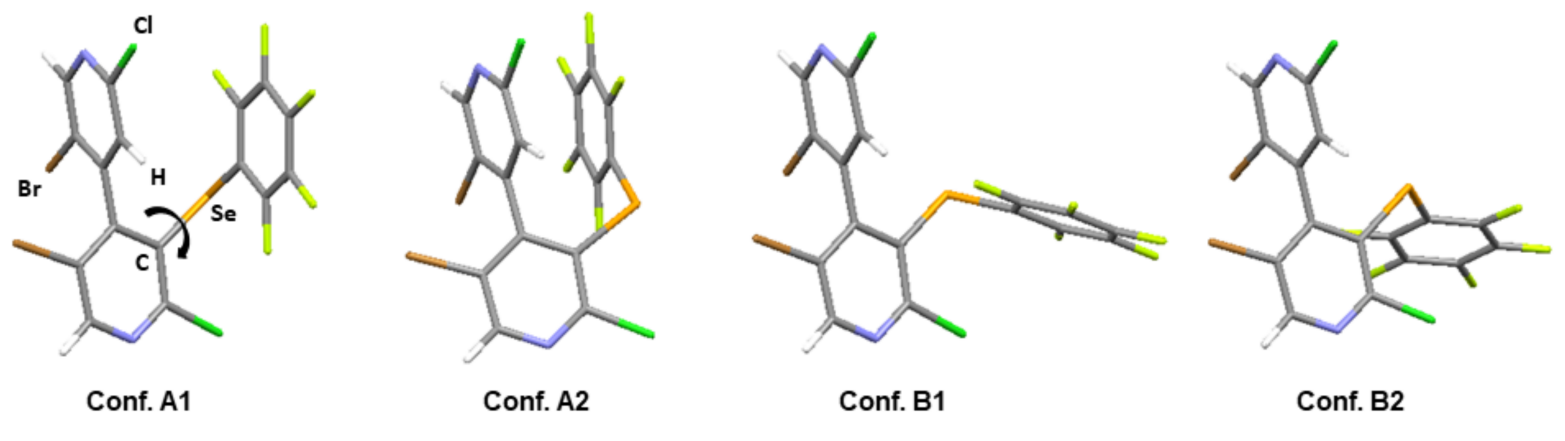
The different conformations calculated for bipyridine **2**.

DFT calculations were run at the CAM-B3LYP-D3/6-311+G(d,p) level of theory to explore the conformation distribution in bipyridines **1** and **2** (Figure 4; for details concerning **6** and **7**, see Figures S3 and S4 in Supplementary Information) focusing on the pentafluorophenyl group orientation. For compounds **1**, **2**, and **6**, four stable conformations were found. They originate from the relative orientation of the pentaflurophenyl ring that can be in front (conformation A) of the upper pyridine ring or away from it (conformation B) due to rotation around the C–Ch bond. For each of the two conformations, two additional conformers are generated by the relative position of the pentafluorophenyl ring, which can be close to the H (conformations A1 and B1) or to the Br (conformations A2 and B2) of the upper pyridine ring.

The relative energies of all conformers were calculated in EtOH using a polarizable continuum model (PCM), allowing for the determination of their relative population (Table 3). The zero value of energy was ascribed to the more stable conformers, that is, Conf. A2 for **1** and Conf. A1 for **2**. We can notice that the distribution of conformers A1, A2, and B1 is different for the two bipyridines, whereas Conf. B2 is negligible in both cases.

**Figure 5:**
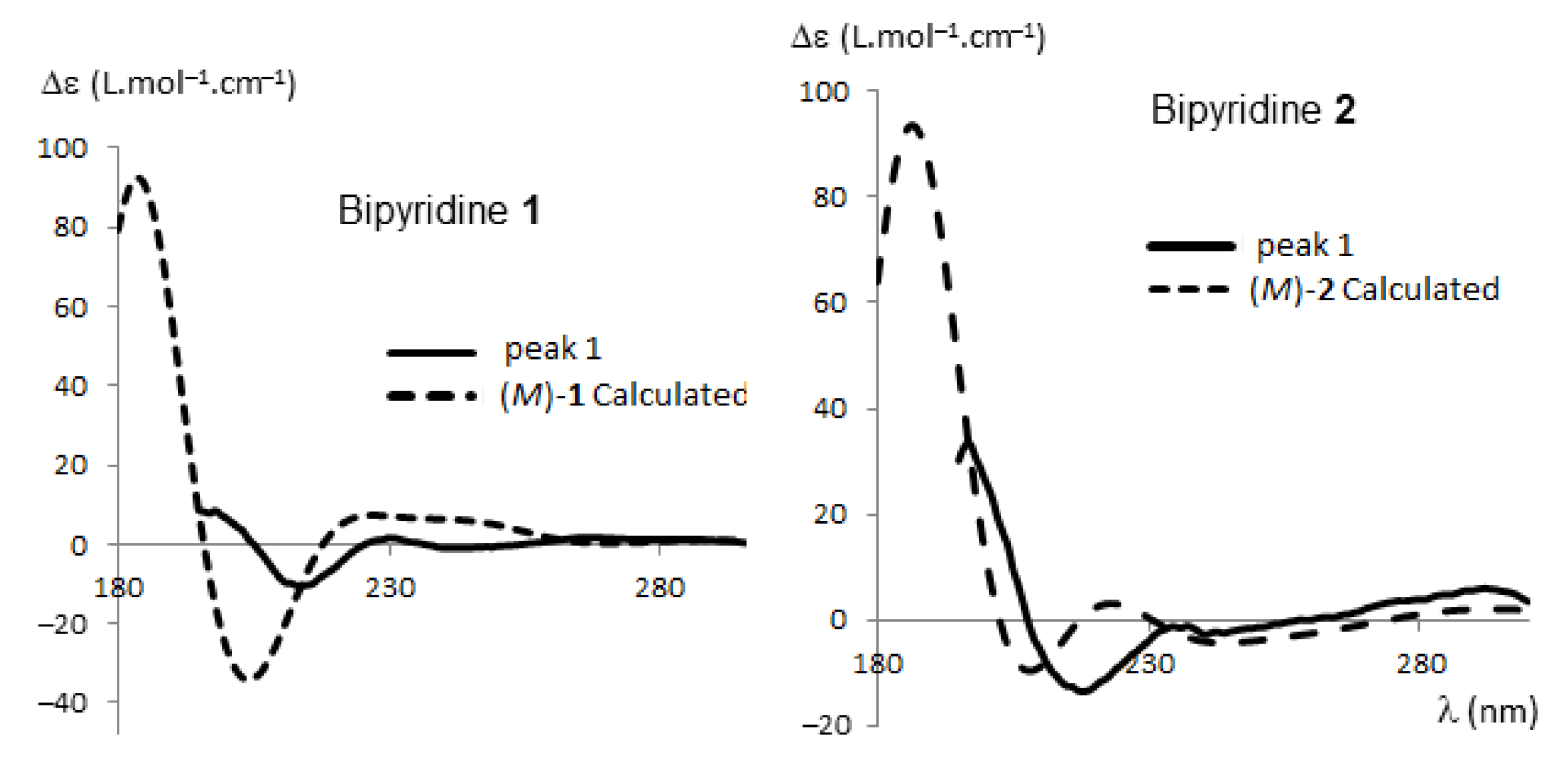
Comparison of measured and calculated ECD spectra for (*M*)-**1** and (*M*)-**2**.

The relative population of each conformer, calculated as Boltzmann statistics at 298.15 K, was taken into account in order to calculate the ECD spectra of (*M*) enantiomers of bipyridines **1**, **2, 6**, and **7**. Comparison of calculated and experimental ECD spectra allowed determining the absolute configurations of the enantiomers (Figure 5; for compounds **6**, **7**, and complete details, see Figures S9–S14 in Supplementary Information). The agreement between experimental ECD of the first eluted enantiomer and theoretical spectra of (*M*)-**1** was good, taking into account the systematic shift of the calculated spectra to shorter wavelengths (10–15 nm), as evidenced in previous studies using the same DFT function [39]. In conclusion, for all bipyridines **1**, **2**, **6** and **7**, peak 1 corresponds to the (*M*) enantiomer and peak 2 to the (*P*) enantiomer. It is worth noting that the (*P*) configuration for peak 2 of compound **6** was confirmed by X-Ray analysis (see Figure S15 in Supplementary Information).

#### 2.2.2. X-Ray Diffraction

**Figure 6:**
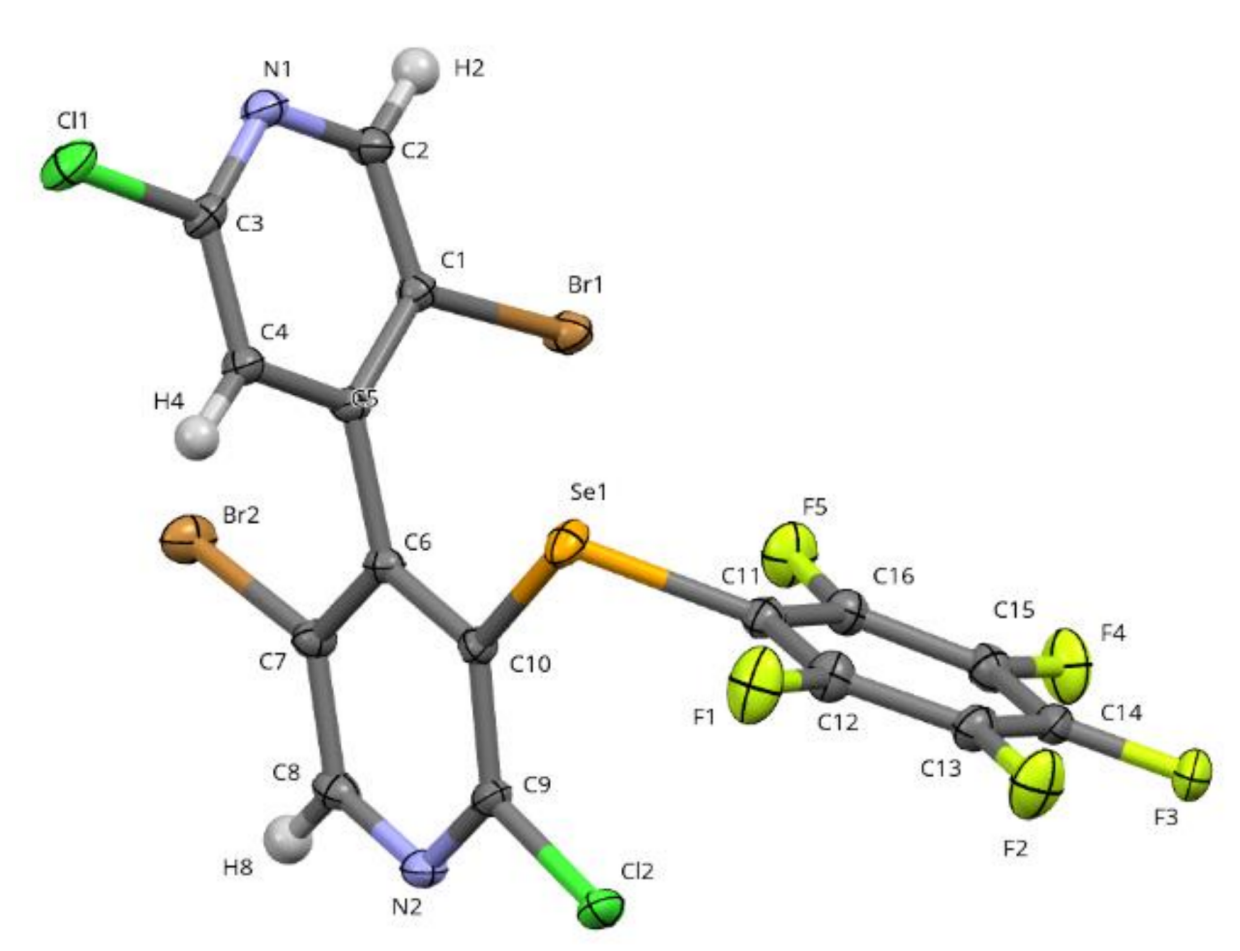
ORTEP plot of **2** with ellipsoids drawn at the 50% probability level.

Compounds **1** and **2** are oily, but after some days in the refrigerator (4 °C), some crystals of racemic **2** could be observed and collected. The X-Ray analysis revealed a single molecule in the asymmetric unit, with a conformation very close to the conformer B2 calculated by DFT (root mean square deviation = 0.16Å) (Figure 6).

The structure of **2** displays various intermolecular interactions. Indeed, molecules are interacting about the inversion center through π···π stacking (centroid–centroid distance of 3.7611(9) Å and slippage of 1.293 Å) and cyclic C4–H4···N2 hydrogen bond (H4···N2 = 2.74Å; C4–H4···N2 = 156°) (Figure S16 in Supplementary Information). A type II halogen···halogen bond is observed where the σ-hole of Cl2(1 − x,y,z) is pointing toward the negative crown of Cl1(x,y,z) (Cl2···Cl1 = 3.349Å; C9–Cl2···Cl1 = 177.14°; Cl2···Cl1–C3 = 101.13°) (Figure S17 in Supplementary Information). The π-hole of the pentafluoro ring is in interaction with the electron rich zone of atoms from three neighboring molecules (N2 = (x,1/2 − y,1/2 + z), N1 = (1 + x,y,z) and the negative crown of Br1 = (1 − x,1 − y,1 − z)) (Figure S18 in Supplementary Information).

#### 2.2.3. ^19^F NMR Spectroscopic Titration with Tetrabutylammonium Chloride (TBACl)

With the aim to probe ChB, binding strength and pattern of bipyridines **1** and **2** with chloride ions were studied in CD_2_Cl_2_ by ^19^F NMR. In addition, to give access to the corresponding binding constants (Ka), this method is expected to give information on the possible competition between the sulfur/selenium σ-holes and the C_6_F_5_ π-hole as electrophilic sites toward chloride

The structures of **1** and **2** contain several atoms (Cl, Br, S/Se) or a group of atoms (C_6_F_5_) able to interact with chloride ions through their σ- or π-hole. Assuming a preferential interaction of S/Se over Cl/Br with σ-hole acceptors [37], we applied the method of Jin and co-workers [43] in order to identify the major interaction (S/Se σ-hole vs C_6_F_5_ π-hole) in compounds **1** and **2**. These authors investigated the σ-hole···Cl^−^ (XB) vs. π-hole···Cl^−^ competition by ^19^F NMR titration of C_6_F_5_X (X = F, Cl, Br, I) in the presence of chloride anion. They observed a direct correlation between Δδ of C_6_F_5_ upon addition of Cl^−^ and the site of interaction. Thus, with C_6_F_6_ and ClC_6_F_5_, which are prone to form π-hole interaction with Cl^−^, the fluorine chemical shifts (δ_F_) moved to lower field. In contrast, with BrC_6_F_5_ and IC_6_F_5_, which preferentially form a XB, δ_F_ moved to higher field.

**Figure 7:**
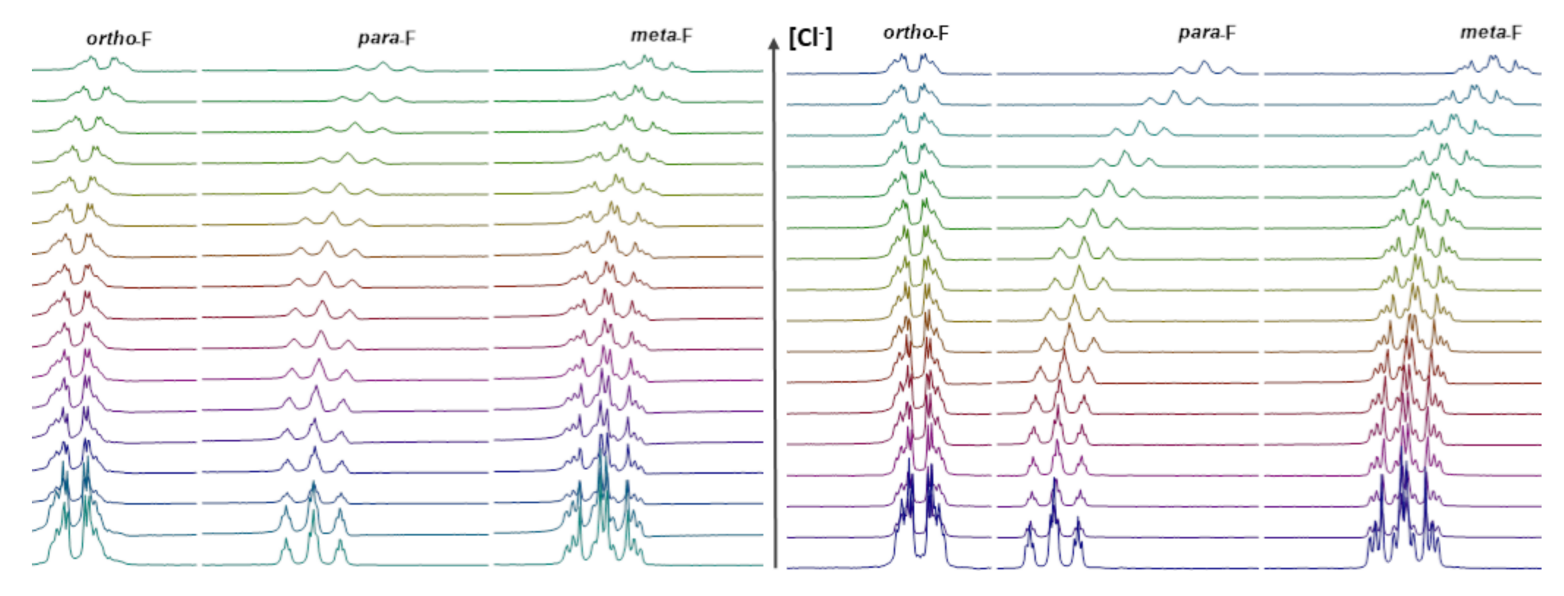
^19^F NMR traces of bipyridines **1** (left) and **2** (right) upon addition of TBACl.

The changes in the ^19^F NMR chemical shift (Δδ) of the ChC_6_F_5_ group were measured by incremental addition of TBACl (from 1 to 200 eq.; see Supplementary Information for details) to a 0.006 mol.L^−1^ solution of **1** and **2** in CD_2_Cl_2_. As shown in Figure 7, δ_F_ of C_6_F_5_ (para-F) in **1** and **2** move to a higher field with the increase in Cl^−^. The variation trends of ortho- and meta-F were lower in both compounds, as previously reported [43]. These results show that both bipyridines **1** and **2** interact preferentially with Cl^−^ through the σ-hole of S and Se, respectively.

**Figure 8:**
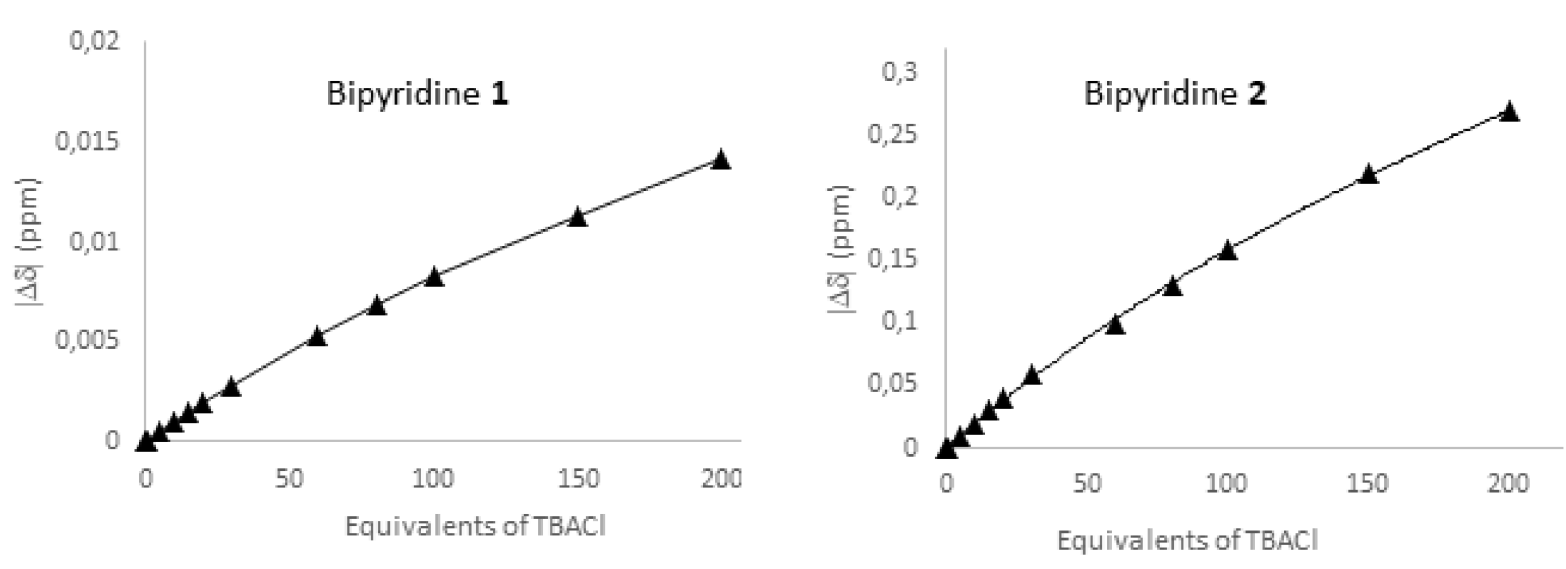
Chemical shift change (Δδ) of para-F in bipyridines **1** and **2** upon addition of TBACl.

For the calculations of Ka, the measured shifts were plotted against the guest equivalents and the resulting curves were fitted using the website calculator http://supramolecular.org/ (Figure 8 and Supplementary Information for details) [44]. By assuming a 1:1 binding model, Ka values of 0.4 M^−1^ and 38 M^−1^ were obtained for compounds **1** and **2**, respectively. It is worth noting that the Ka value for **2** (38 M^−1^) is similar to the one obtained by Matile and coworkers with bis(pentafluorophenyl)selenium [17].

### 2.3. Catalysis

The hydrogen-transfer reaction using Hantzsch ester (HE) as hydrogen source is a mild method to reduce quinolines and other C=N substrates [45]. Brønsted acids are known to be particularly good catalysts to promote this reaction [46]. In 2008, Bolm showed that haloperfluoroalkanes can be used as XB catalysts for the reduction of 2-phenylquinoline **10** using HE [47]. Since then, this reduction has often been used as a benchmark reaction to evaluate the activity of XB [48,49] and ChB [15,16] catalysts.

We therefore selected the reduction of 2-phenylquinoline **10** as a model substrate for investigating the catalytic ability of the ChB donors **1** and **2** (Table 4). The reaction was followed by ^1^H NMR over time and it was stopped after 72 h, even if complete conversion was not reached. The resulting tetrahydroquinoline **11** was only isolated and the yield given, when the reaction was completed.

Without catalyst, no conversion was observed after 72 h (entry 6). Rewardingly, with 20 mol% of **1** or **2** as catalysts, the reaction was complete after 48 h, delivering the fully reduced compound **11** with 87% and 94% yields, respectively (entries 1 and 2). Interestingly, with only 5 mol% of **1**, a good conversion of 81% was still obtained after 72 h of reaction (entry 3). Under the same conditions, catalyst **2** was even more active, giving full conversion after 72 h, and compound **11** was obtained with an isolated yield of 88% (entry 4). It is interesting to note that with **2**, the reaction reached 54% conversion only after 18 h (entry 5).

In the presence of 1 eq. of TBACl, the conversion was lower but not null (entry 7), showing that probably the σ-hole of bromine atoms and/or the π-hole of the C_6_F_5_ group took the relay in the catalytic activation of the quinoline substrate. In order to verify this hypothesis, compounds **12** [38] and **13** [37] were tested as catalysts in the reaction (entries 8 and 9). Even with 20 mol%, the reaction did not reach completion after 72 h, showing that the Se atom in compound **2** is the primary site of interaction with the substrate. However, the 62% conversion obtained with **12** is an indication that bromine atoms alone can activate the substrate (entry 8). The presence of the C_6_F_5_ group in **13** compared to **12** slightly increased the conversion from 62 to 78%, a result which revealed the modest contribution of the π-hole in the substrate activation (entry 9). With the iodinated compound **3**, complete conversion was reached only after 72 h, showing the beneficial effect of the SeC_6_F_5_ group compared to iodine atom (entries 10 and 11). Finally, the (*P*) enantiomer of **2** was employed as catalyst, but unfortunately, no asymmetric induction was observed (entry 12).

## 3. Discussion

**Figure 9:**
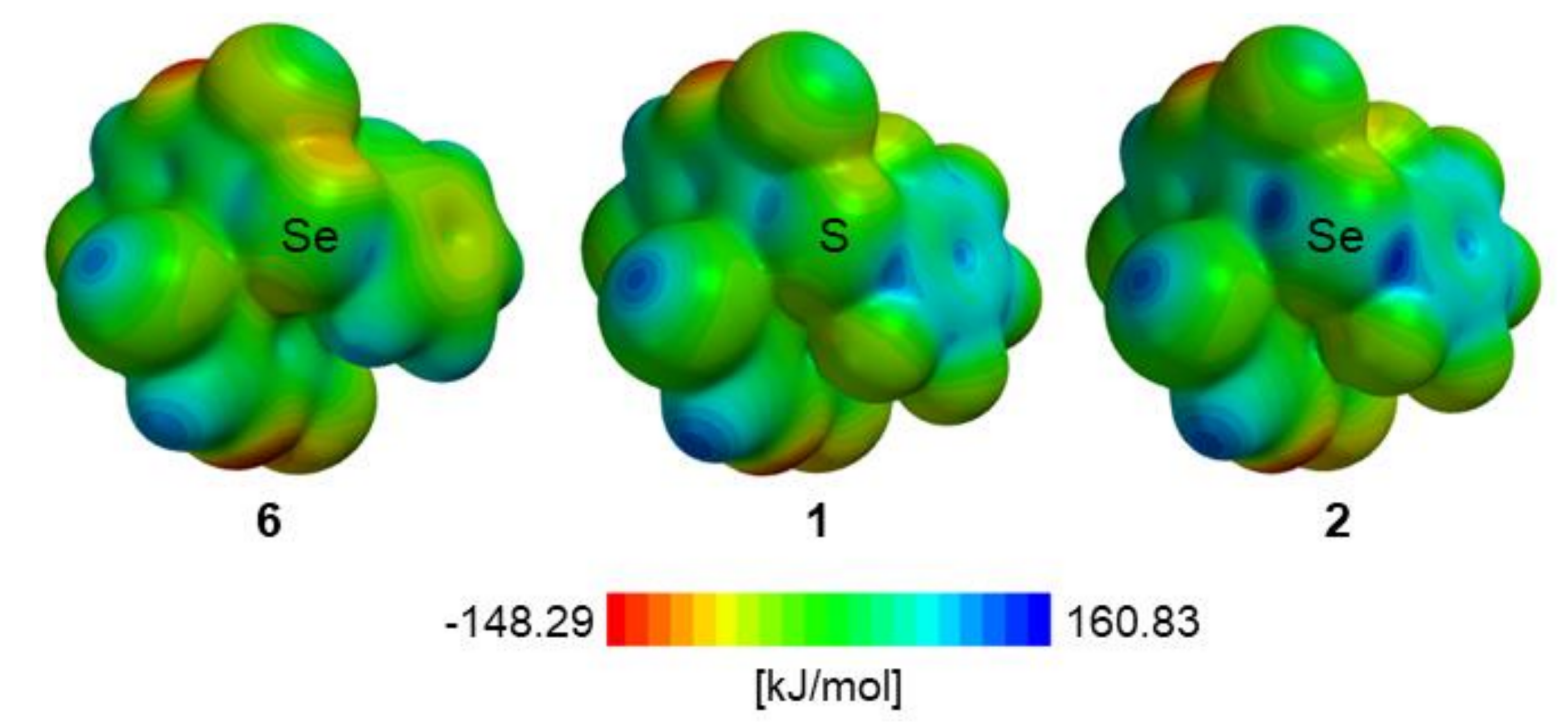
Ab initio electrostatic potential surfaces (EPS) of bipyridines **6**, **1**, and **2.**

The new motif, which **1** and **2** are based on, was designed by introducing on the Ch site two electron withdrawing groups (EWGs), namely, a pentafluorophenyl ring and a tetrahalo-4,4′-bipyridine scaffold. Indeed, computation of electrostatic potential (EP) maxima on the Ch site indicates that fluorination increase σ-hole depth [50]. In our study, the ChB donor ability ranking for conformationally related (Figure 4, A1-type) structures of 5,5′-dibromo-2,2′-dichloro-3-chalcogeno-4,4′-bipyridines **6**, **1**, and **2** was determined by evaluating the EP maxima on calculated EP surfaces (DFT/B3LYP/6-311G*), proving that EP maxima values on the Ch σ-hole increase upon fluorination (max EP_Ch_: SePh (**6**) < SC_6_F_5_ (**1**) < SeC_6_F_5_ (**2**)) (Figure 9 and Table 5). As expected, σ-hole depth increases with the polarizability of the Ch atom. Moreover, the pentafluorophenyl moiety is a well-known π-hole center [51]. On the other hand, the tetrahalo-4,4′-bipyridine skeleton contributes to increase the electrophilic character of the Ch atom and makes the new motif chiral, due to its atropisomeric geometry. In contrast, most reported ChB donors are characterized by a charged heterocyclic structure, which provides sufficient polarization to the Ch atom [2,3,14]. Another peculiarity of the new motif described here is the possibility of rotation around the C–Ch bond, which offers conformational adjustments/tuning during catalysis. It is worth noticing that establishing strong ChBs requires angles R-Ch∙∙∙B_L_ close to 180°, and thus, conformational flexibility of the ChB donor catalyst should increase its catalytic efficiency. In this regard, it is worth noting that, in the compounds so far studied as organocatalysts, the Ch sites are located in a rigid heteroaromatic structure [15,16,17,18,19,20,21].

On this basis, sulfur- and selenium-based chiral catalysts **1** and **2** were prepared by two different methods from chiral 5,5′-dibromo-2,2′-dichloro-3-iodo-4,4′-bipyridine **3**. The first method, based on a iodine–lithium exchange followed by quenching with bis(pentafluorophenyl)dichalcogenide, delivered the expected products with low yields. Higher yields were obtained by a copper-mediated coupling of pentafluorophenylthiolate and selenolate with **3**. This method proved to be very efficient and selective for the iodinated position. It is therefore expected to proceed with comparable efficiency with other chiral polyhalogenated 4,4′-bipyridines [42] and could represent a new entry to various chiral chalcogen compounds.

The enantiomers of **1** and **2** were separated by HPLC on chiral stationary phase and their absolute configuration was assigned by comparison of the measured and calculated ECD spectra. ECD analysis showed that 5,5′-dibromo-2,2′-dichloro-3-(perfluorophenyl)chalcogeno- 4,4′-bipyridines are present in solution as a mixture of four conformers because of the rotation of the pentafluorophenyl group along the C–Ch bond. Unfortunately, our efforts to detect these conformers in solution by variable temperature ^1^H NMR were unsuccessful. We could, however, isolate few crystals of the selenium derivative **2** (as a racemate) at low temperature and their XR structure only showed the presence of the minor conformer **B2**, as revealed from the calculations in solution (Figure 4).

The ability of the chalcogen compounds to drive σ-hole-based interactions in solution was studied through ^19^F NMR by incremental addition of chloride ions serving as strong acceptors. We observed that δ_F_ of ChC_6_F_5_ move to a higher field with increasing chloride concentration, showing that both bipyridines interact preferentially with Cl^−^ through the σ-hole of S and Se. Moreover, this study allowed obtaining the binding constants values (Ka) of the 1:1 adducts between compounds **1** or **2** and chloride ion. 

**Scheme 2:**
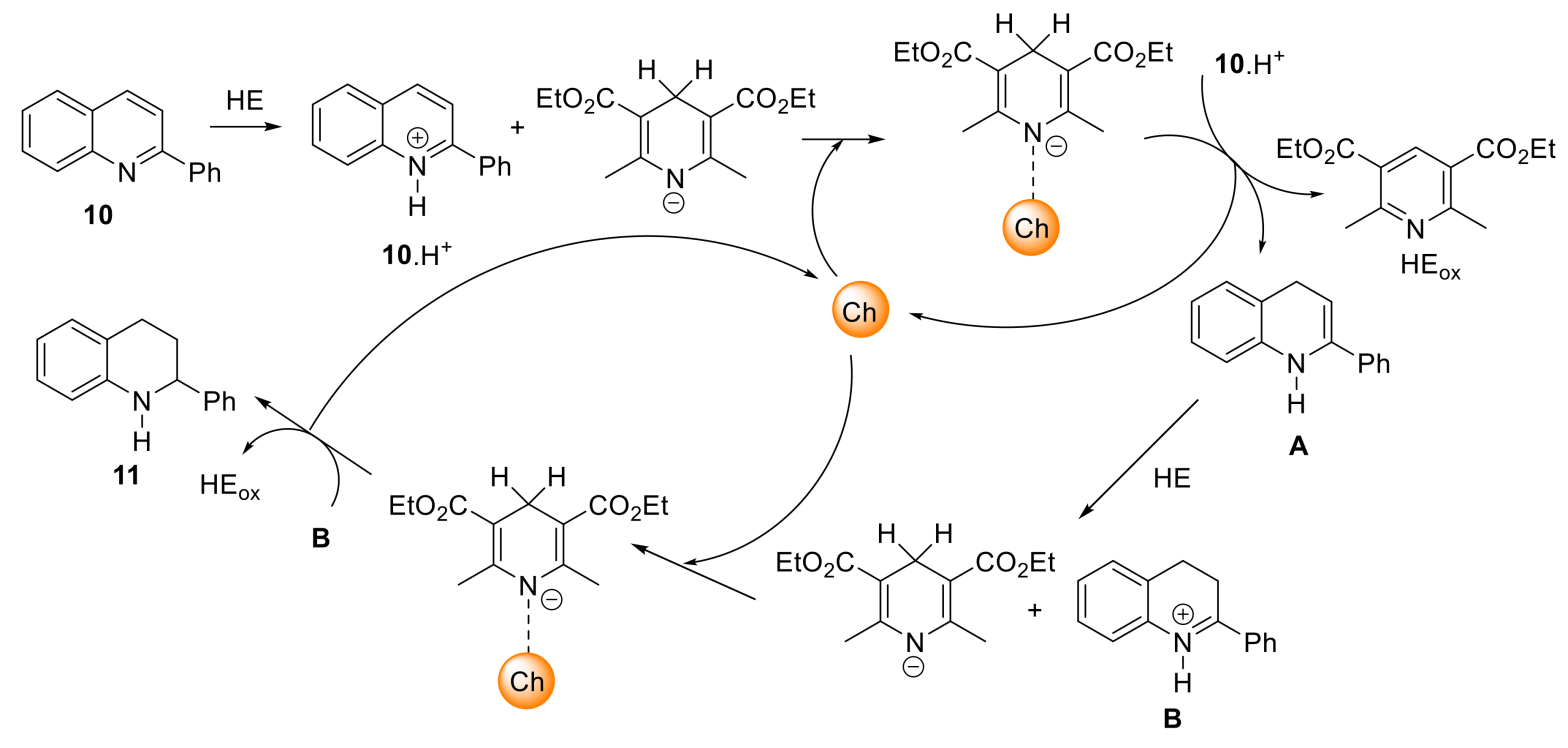
Possible mechanism for the ChB-catalyzed reduction of 2-phenylquinoline **10**.

After having shown that derivatives **1** and **2** can behave as ChB donors in solution, their catalytic activity was evaluated in the reduction of 2-phenylquinoline **10**. As expected from the Ka values, the Se compound **2** was more active than its S analog **1**. Therefore, the study was mainly focused on the use of **2** in comparison with reference compounds where the SeC_6_F_5_ group was replaced by CH_3_ (**12**), CH_2_C_6_F_5_ (**13**), and I (**3**). The reference compound **12** without a σ- or π-hole donor in 3-position was chosen so as to keep the torsional angle between the two pyridine rings similar to compound **2** in order to conserve the same geometry and to analyze only the effect due to the presence of the SeC_6_F_5_ group. In agreement with the study in solution, the catalysis results show that **2** interacts in priority through the Se atom with the substrate. It is worth noting that other sites can interact with the substrate, although to a lesser extent: The two bromine atoms in 5,5′-position and the C_6_F_5_ group. The last aspect concerns the absence of enantioselectivity when using enantiomerically pure **(*P*)-2**. This result may be due to the complex mechanism of the selected reaction (Scheme 2).

After protonation of quinoline **10** by Hantzsch ester (HE), the first reduction occurs to give enamine intermediate **A.** The latter is protonated to **B** and then further reduced by the second equivalent of HE. Both reductions can be activated by the ChB donor catalyst. A recent DFT study by Wang et al. with a XB donor catalyst showed that in both reductions, the halogen atom interacts with the deprotonated HE, thus facilitating the hydride transfer [52]. If the same activation mode occurs with catalyst **(*P*)-2** through ChB, the chiral bipyridine backbone would be placed far from the C=N bond of the substrate, and such spatial arrangement could explain the lack of asymmetric induction.

On these bases, we are currently working on the synthesis of analogs of **2**, where bromines are exchanged by chlorines or fluorines and the C_6_F_5_ group by a CF_3_. Furthermore, the search for new ChB-catalyzed reactions, for which asymmetric induction could be achieved, are currently underway in our laboratory.

## 4. Materials and Methods

### 4.1. General Information

Proton (^1^H NMR), carbon (^13^C NMR), fluorine (^19^F NMR), and selenium (^77^Se NMR) nuclear magnetic resonance spectra were recorded on a Bruker Avance III instrument operating at 300, 400, or 500 MHz (Bruker Corporation, Billerica, MA, USA). The chemical shifts are given in parts per million (ppm) on the delta scale. The solvent peak was used as reference values for ^1^H NMR (CDCl_3_ = 7.26 ppm) and for ^13^C NMR (CDCl_3_ = 77.16 ppm). An external standard was used for ^19^F NMR (trifluoromethylbenzene) and for ^77^Se NMR (diphenyl diselenide). Data are presented as follows: Chemical shift, multiplicity (s = singlet, d = doublet, t = triplet, q = quartet, quint = quintet, m = multiplet, b = broad), integration, and coupling constants (J/Hz). High-resolution mass spectra (HRMS) data were recorded on a micrOTOF spectrometer (Bruker Corporation, Billerica, MA, USA) equipped with an orthogonal electrospray interface (ESI). Analytical thin layer chromatography (TLC plates from Merck KGaA, Darmstadt, Germany) was carried out on silica gel 60 F254 plates with visualization by ultraviolet light. Reagents and solvents were purified using standard means. Tetrahydrofuran (THF) was distilled from sodium metal/benzophenone and stored under an argon atmosphere. Anhydrous reactions were carried out in flame-dried glassware and under an argon atmosphere. Bipyridine **3** [38] and bis(perfluorophenyl)diselenide **5a** [53] were prepared according to the literature. All other chemicals were used as received. An Agilent Technologies (Waldbronn, Germany) 1100 Series HPLC system (high-pressure binary gradient system equipped with a diode-array detector operating at multiple wavelengths (220, 254, 280 nm), a programmable autosampler with a 20 μL loop, and a thermostated column compartment) was employed for multimilligram separations. Data acquisition and analyses were carried out with Agilent Technologies ChemStation Version B.04.03 chromatographic data software. The UV absorbance is reported as milliabsorbance units (mAU). Chiralpak IA (immobilized amylose tris-3,5-dimethylphenylcarbamate) and Chiralpak IC (immobilized cellulose tris-3,5-dichlorophenylcarbamate) were used as chiral columns (250 × 4.6 mm) (5 μm) (Chiral Technologies Europe, Illkirch, France). HPLC-grade *n*-hexane (Hex) and 2-propanol (IPA) were purchased and used as received. CD spectra were recorded on a « J-810 » from Jasco (JASCO International Co. Ltd., Tokyo, Japan) at room temperature using 0.15–0.30 mM samples in ethanol and a 1 mm quartz cell, with the following conditions: 100 nm/min scanning speed, 1 nm data pitch, 4.0 nm bandwidth, 1 s response time.

### 4.2. General Procedure for the Synthesis of **2**, **6**, and **7** from Bipyridine **3**

Bipyridine **3** (0.19–0.47 mmol) was dissolved in THF (5–8 mL) and the solution was cooled to −78 °C. *n*-BuLi (1.2 M in hexanes, 1.01 eq.) was added dropwise and the mixture was stirred at −78 °C for 1 h. A solution of diselenide **5a**–**c** (2.5 eq.) in THF (2–5 mL) was slowly added and stirring was maintained at −78 °C for 30 min. The temperature was raised to room temperature during 5 h before addition of water (10 mL). The mixture was extracted with ethyl acetate (4 × 10 mL); the organic phases were combined, washed with brine (30 mL), and dried over anhydrous Na_2_SO_4_. After concentration, the crude was purified by chromatography on silica gel (20% dichloromethane/pentane) and, if necessary, a flash column chromatography was used to remove impurities (2% ethylacetate/cyclohexane).

*5,5′-Dibromo-2,2′-dichloro-3-((perfluorophenyl)selanyl)-4,4′-bipyridine (**2**).* Colorless oil (46 mg, 34%). R_f_ = 0.48 (40% dichloromethane/pentane). ^1^H NMR (500 MHz, CD_2_Cl_2_) δ 8.66 (s, 1H); 8.62 (s, 1H); 7.07 (s, 1H). ^13^C NMR (126 MHz, CD_2_Cl_2_) δ 155.1; 152.7; 152.6; 152.6; 151.2; 149.7; 148.6–145.8 (m); 144.1–141.2 (m); 139.5–136.8 (m); 126.1, 125.1 (d, *J* = 1.3 Hz); 120.3; 119.9; 102.6 (t, *J* = 23.3 Hz). ^19^F NMR (471 MHz, CD_2_Cl_2_) δ −127.0 to −127.1 (m); −150.7 to −150.8 (m); −159.8 to −160.0 (m). ^77^Se NMR (114 MHz, CD_2_Cl_2_) δ 285.2. HRMS (ESI–TOF) [M + H]^+^ *m/z*: Calcd. for C_16_H_4_Br_2_Cl_2_F_5_N_2_Se 626.7198, found: 626.7180.

*5,5′-Dibromo-2,2′-dichloro-3-(phenylselanyl)-4,4′-bipyridine (**6**).* Colorless oil (54 mg, 20%). R_f_ = 0.29 (5% ethylacetate/cyclohexane). ^1^H NMR (500 MHz, CDCl_3_) δ 8.62 (s, 1H); 8.54 (s, 1H); 7.31–7.26 (m, 1H); 7.21 (t, *J* = 7.6 Hz, 2H); 7.17–7.12 (m, 2H); 6.71 (s, 1H). ^13^C NMR (126 MHz, CDCl_3_) δ 155.5; 152.7; 152.0; 151.4; 150.3; 149.5; 132.8; 129.9; 129.7; 129.0; 128.6; 125.1; 119.7; 119.2.^77^Se NMR (114 MHz, CDCl_3_) δ 425.2. HRMS (ESI–TOF) [M + H]^+^ *m/z*: Calcd. for C_16_H_8_Br_2_Cl_2_N_2_Se 536.7669, found: 536.7645.

*5,5′-Dibromo-2,2′-dichloro-3-(methylselanyl)-4,4′-bipyridine (**7**).* Colorless oil (48 mg, 51%). R_f_ = 0.56 (5% ethylacetate/cyclohexane). ^1^H NMR (500 MHz, CD_2_Cl_2_) δ 8.65 (s, 1H), 8.59 (s, 1H); 7.13 (s, 1H); 2.31 (s, 3H). ^13^C NMR (126 MHz, CD_2_Cl_2_) δ 155.9; 152.8; 152.0; 150.9; 150.9; 150.5; 128.2; 124.6; 119.2; 118.6; 10.0. ^77^Se NMR (114 MHz, CDCl_3_) δ 459.0. HRMS (ESI-TOF) [M + H]^+^ *m/z*: Calcd. for C_11_H_7_Br_2_Cl_2_N_2_Se 474.7513, found: 474.7487.

### 4.3. General Procedure for the Copper-Mediated Synthesis of **1**, **2**, and **8**

Under argon atmosphere, a 25 mL Schlenck tube was filled with copper powder (2 eq.) and anhydrous NMP (1–3 mL). Bis(pentafluorophenyl)dichalcogenide (1.3 eq.) was added and the mixture stirred for 3 h at room temperature. Bipyridine **3** (1 eq.) was added and the mixture was stirred overnight at a given temperature (100 or 130 °C). The reaction was allowed to cool to room temperature, diluted with diethyl ether (30 mL), and filtered. The filtrate was washed with brine (4 × 15 mL). The organic phase was dried over Na_2_SO_4_, evaporated and the residue was submitted to two successive columns chromatography on silica gel (5% diethyl ether/pentane for the first one and 2% diethyl ether/pentane for the second).

Bipyridine **1** [37] was obtained as a colorless oil (85 mg; 74%) by using the following conditions (Table 1, entry 2): Cu powder (24.8 mg, 0.39 mmol), bis(pentafluorophenyl)disuldife **4** (103.5 mg, 0.26 mmol), bipyridine **3** (100.3 mg, 0.197 mmol), at 100 °C.

Bipyridine **2** was obtained as a colorless oil (245 mg; 81%) by using the following conditions (Table 1, entry 11): Cu powder (63.5 mg, 1.0 mmol), bis(pentafluorophenyl)diselenide **5a** (251 mg, 0.51 mmol), bipyridine **3** (200.5 mg, 0.394 mmol), at 100 °C.

*5′-Bromo-2,2′-dichloro-3,5-bis((perfluorophenyl)thio)-4,4′-bipyridine (**8**)* (Table 1, entry 3). It was obtained as a colorless oil (16 mg, 39%) by using the following conditions: Cu powder (9.5 mg, 0.15 mmol), bis(pentafluorophenyl) disuldife **4** (30.6 mg, 0.077 mmol), bipyridine **3** (30 mg, 0.059 mmol), at 130 °C. ^1^H NMR (500 MHz, CDCl_3_) δ 8.59 (s, 1H); 8.47 (s, 1H); 7.12 (s, 1H). ^13^C NMR (125 MHz, CDCl_3_) δ 155.2; 152.4; 152.2; 151.8; 151.0; 148.4–147.5 (m); 146.6; 146.4–144.9 (m); 140.08–135.52 (m); 128.6; 127.0, 124.6; 119.8; 106.9–105.4 (m); 106.8–105.6 (m). ^19^F NMR (471 MHz, CDCl_3_) δ −131.1 to −131.3 (m); −132.3 to −132.4 (m); −148.1 (t, *J* = 20.9 Hz); −150.0 (t, *J* = 20.8 Hz); −158.4 to −158.7 (m); −159.0 to −159.4 (m). HRMS (EI^+^) [M]^+^ *m/z*: Calcd. for C_22_H_3_BrCl_2_N_2_S_2_ 698.8257, found: 698.8211.

### 4.4. General Procedure for the Catalytic Reduction of 2-Phenylquinoline **10**

2-Phenylquinoline **10** (9.5 mg, 49 μmol), Hantzsch ester (27.1 mg, 107 μmol) and hexamethylbenzene as internal standard (δ 2.23 ppm, s, 18H), were weighted in to a screw cap vial and suspended in dry degassed CD_2_Cl_2_ (1 mL). Then, the desired amount of catalyst (5 or 20 mol%) was added from a stock solution of catalyst (120 mM). The vial was tightly sealed and stirred at 25 °C. ^1^H NMR spectra of aliquots of the reaction mixture (5–10 μL) diluted in CDCl_3_ (0.5 mL) were recorded at varying time intervals. When the reaction was finished or at 72 h maximum, the solvent was removed under reduced pressure and the remaining product was purified by flash column chromatography on silica gel (3% ethyl acetate/pentane) to afford tetrahydroquinoline **11**.

### 4.5. Crystal Data for **2**

C_16_H_3_Br_2_Cl_2_F_5_N_2_Se, M = 627.88, monoclinic, a = 10.9949(5) Å, b = 13.7036(6) Å, c = 12.1716(6) Å, V = 1827.20(15) Å^3^, *T* = 120(2) K, space group P2_1_/c, Z = 4, μ (Mo Kα) = 6.775 mm^−1^, 74843 reflections measured, 6383 independent reflections (R_int_ = 0.0467). The final R_1_ value were 0.0221 (I > 2σ(I)) and 0.0240 (all data). The final w_R_(F^2^) values were 0.0552 (I > 2σ(I)) and 0.0560 (all data). The goodness of fit on F^2^ was 1.117. CCDC no. 1963859.

## Figures and Tables

**Table 1:**
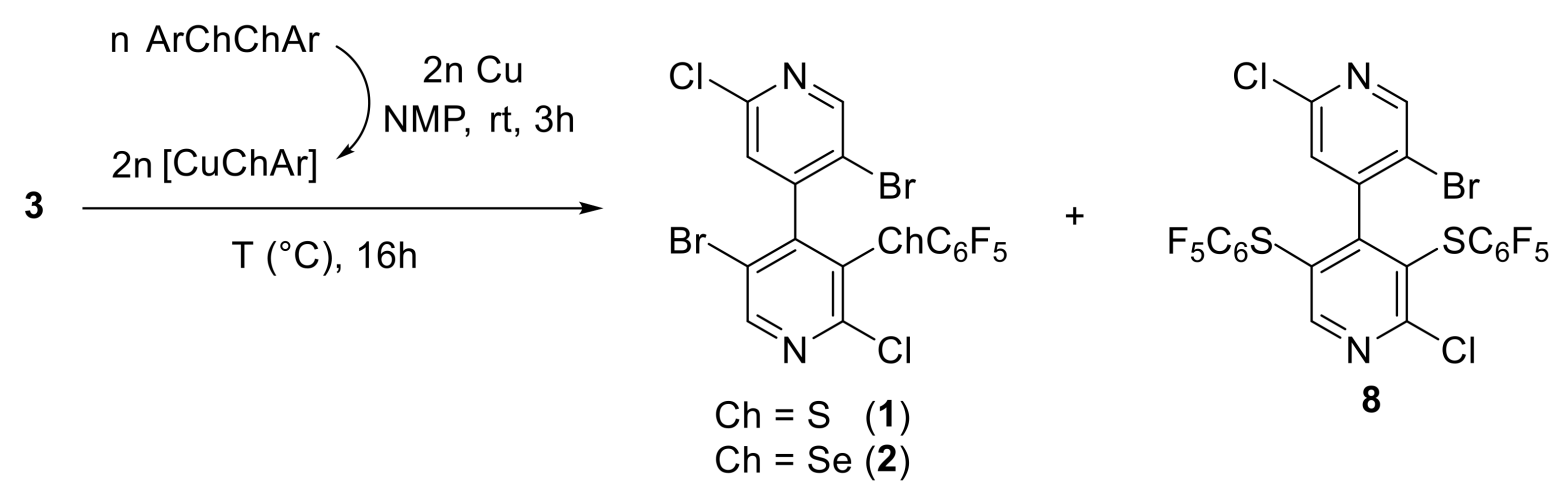
Synthesis of **1** and **2** through copper-mediated cross-coupling reaction.

Entry	ArChChAr	n (eq.)	T (°C)	NMR Ratio 3/1/8 or 3/2	Yield (%)
1	(C_6_F_5_S)_2_ (**4**)	1.3	80	33/67/0	−
2	‘’	1.3	100	12/77/11	74 (**1**)
3	‘’	1.3	130	4/31/65	39 (**8**)
4	‘’	0.5	100	78/20/2	−
5	‘’	0.8	100	53/43/4	−
6	‘’	1.0	100	39/57/4	−
7	‘’	2.0	100	0/75/25	67 (**1**)
8	‘’	4.0	100	0/54/46	
9	‘’	0.5	130	49/42/9	−
10^a^	‘’	1.3	100	17/63/20	−
11	(C_6_F_5_Se)_2_ (**5a**)	1.3	100	14/86	81 (**2**)

^a^ The reagents were mixed together without preforming the copper complex.

**Table 2:** Optimized multimilligram enantioseparation of 4,4′-bipyridines **2**, **6**, and **7** ^a^.

Bipyridine	Racemate [mg]	Absolute Configuration (e.e.^b^ %)		Recovered Amounts [mg] (Recovery%)	
		1st Eluted Peak	2nd Eluted Peak	1st Eluted Peak	2nd Eluted Peak
**2**	30.0	>99	98.8	10.6 (70.7)	13.0 (86.7)
**6**	40.0	>99	95.0	7.4 (37.0)	18.0 (90.0)
**7**	12.0	>99	95.1	5.1 (85.0)	5.9 (98.3)

^a^ Mobile Phase: n-Hexane/2-propanol 90:10, flow rate 0.5 mL/min, T = 22 °C. ^b^ Enantiomeric excess (e.e.) determined by chiral HPLC under the same conditions used for recoveries.

**Table 3:** Relative energies and populations of the different conformers of **1** and **2**.

	1				2			
	A1	A2	B1	B2	A1	A2	B1	B2
ΔG (kJ/mol)	0.66	0	0.74	8.09	0	3.17	0.96	7.53
Pop. (%)	30	39	29	2	50	14	34	2

**Table 4:**
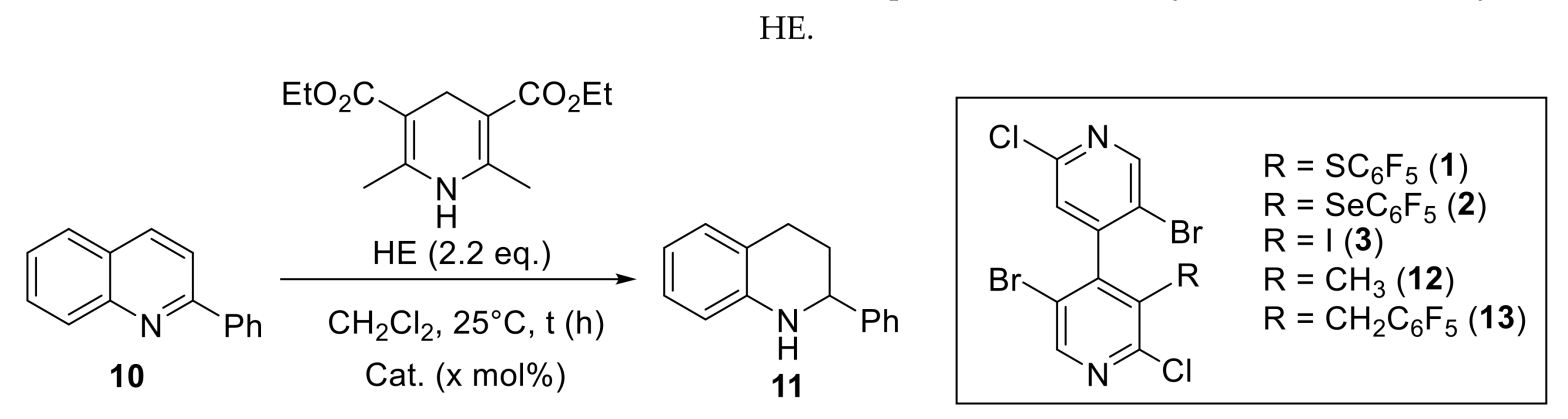
Effect of ChB donors **1** and **2** and reference compounds on the catalytic reduction of **10** by HE.

Entry	Cat.	x (mol%)	Time (h)	Conv. (%)^a^	Yield (%)
1	**1**	20	48	100	87
2	**2**	20	48	100	94
3	**1**	5	72	81	−
4	**2**	5	72	100	88
5	**2**	5	18	54	−
6	-	-	72	0	−
7^b^	**2**	5	18	38	−
8	**12**	20	72	62	−
9	**13**	20	72	78	−
10	**3**	20	48	68	−
11	**3**	20	72	100	86
12	**(*P*)-2**	20	48	100	90 (0% ee)

^a^ Calculated by ^1^H NMR with hexamethylbenzene as internal standard. ^b^ 1 eq. of TBACl was added.

**Table 5:** Electrostatic potential (EP) maxima [kJ/mol] on Ch σ-holes for compounds **1**, **2**, and **6** ^a^.

**σ-hole ^b^**	**6**	**1**	**2**
1	88.0	113.2	136.7
2	73.6	98.6	127.5

^a^ Spartan ’10 Version 1.1.0 (Wavefunction Inc., Irvine, CA), isovalue 0.002. ^b^ σ-hole (1) is located on the elongation of the C–Ch bond; σ-hole (2) is located on the elongation of the C_ArF_–Ch bond.

## Supplementary Materials

The following are available online at https://www.mdpi.com/1420-3049/24/24/4484/s1: Figures S1 and S2: ECD spectra of **6** and **7**; Figures S3 and S4: Calculated conformations for **6** and **7**; Figures S5–S8: Comparison of measured and calculated UV spectra; Figures S9–S12: Calculated ECD spectra for each conformer of **(*M*)-1**, **(*M*)-2**, **(*M*)-6** and **(*M*)-7**; Figures S13 and S14: Comparison of measured and calculated ECD spectra for **(*M*)-6** and **(*M*)-7**; Figure S15: ORTEP plot of **(*P*)-6**; Tables S1–S5: X-Ray details for bipyridine **2**; Figures S16–S18: Intermolecular interactions in the X-Ray structure of **2**, DFT details; Table S6: Anion binding experiments: Overview of host addition; Table S7: Binding constants Ka for **1** and **2**; Figures S19–S34: NMR spectra.
