# Supplementary material for: Chiral Chalcogen Bond Donors Based on the 4,4′-Bipyridine Scaffold
Source: Molecules. 2019 Dec 6;24(24):4484. doi: 10.3390/molecules24244484 (PMC6943643; doi:10.3390/molecules24244484)
Supplement: Supplementary file 1 [file molecules-24-04484-s001.pdf]

Supporting Information

Chiral chalcogen bond donors based on the 4,4'-bipyridine scaffold

Robin Weiss [1], Emmanuel Aubert [2], Paola Peluso [3,*], Sergio Cossu [4], Patrick Pale [1] and Victor Mamane [1,*]

[1] *Institut de Chimie de Strasbourg, UMR CNR 7177, Equipe LASYROC, 1 rue Blaise Pascal, 67008 Strasbourg Cedex, France.*

[2] *Cristallographie, Résonance Magnétique et Modélisations (CRM2), UMR CNRS 7036, Université de Lorraine, Bd des Aiguillettes, 54506 Vandoeuvre-les-Nancy, France.*

[3] *Istituto di Chimica Biomolecolare ICB, CNR, Sede secondaria di Sassari, Traversa La Crucca 3, Regione Baldinca, 07100 Li Punti, Sassari, Italy.*

[4] *Dipartimento di Scienze Molecolari e Nanosistemi DSMN, Università Ca' Foscari Venezia, Via Torino 155, 30172 Mestre Venezia, Italy.*

* *Correspondence: vmamane@unistra.fr; Tel.: +33 3 68851612 (VM). paola.peluso@cnr.it; Tel.: +39 079 2841218 (PP).*

Table of Contents

I. UV spectra, ECD spectra and conformational analyses (Fig. S1-S14)…….………..S2

II. X-Ray diffraction analysis (Table S1-S5 & Fig. S15-S18).……………………………S9

III. DFT details………………………………………..…………..…………………S22

IV. Anion binding experiments (Table S6-S7)……..……………………………………S23

V. NMR spectra (Fig. S19-S34)………………..………………………………………..S24

I. ECD spectra and conformational analyses

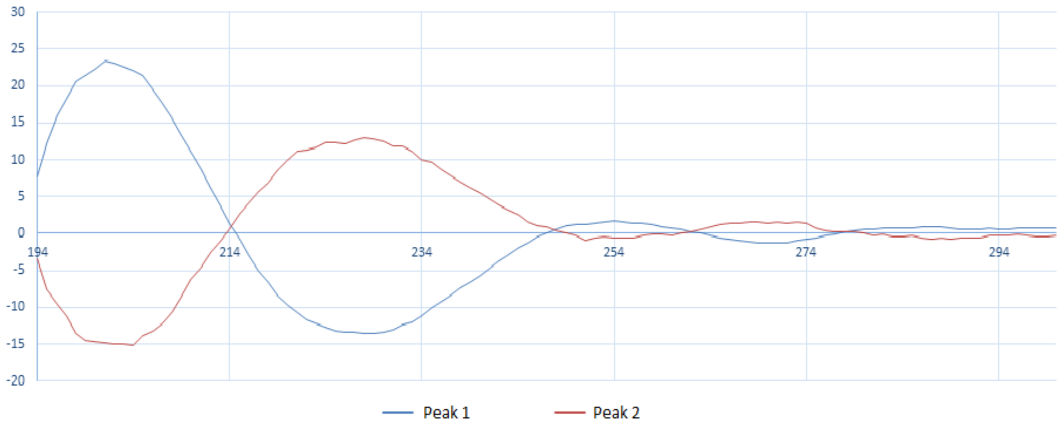

Fig. S1. ECD spectra of the enantiomers of bipyridine **6**

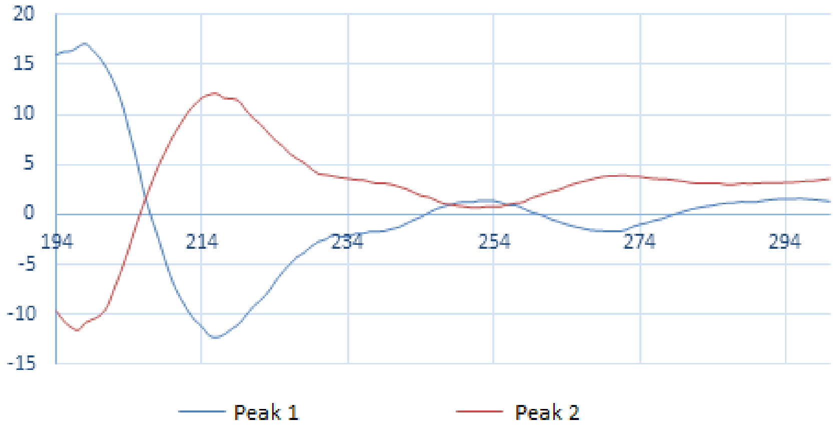

Fig. S2. ECD spectra of the enantiomers of bipyridine **7**

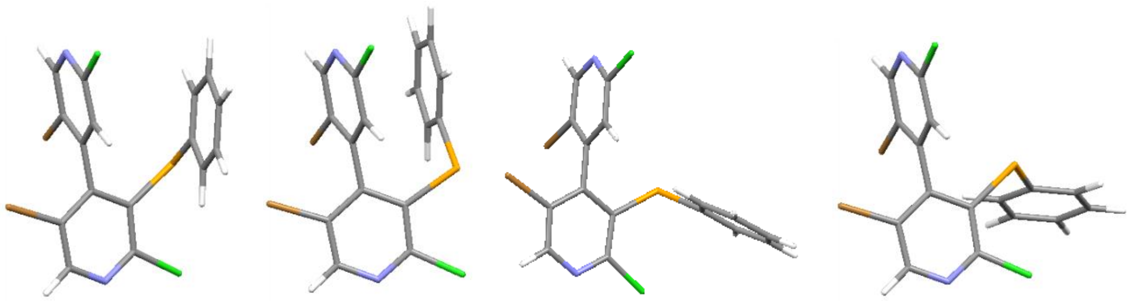

Conf.	A1	A2	B1	B2
ΔG (kJ/mol)	0	7.54	4.99	7.47
Pop. (%)	81	4	11	4

Fig. S3. The different conformations calculated for bipyridine **6** in ethanol

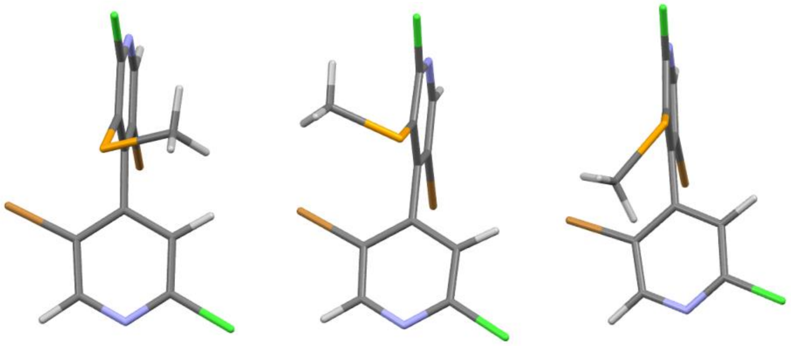

Conf.	A1	A2	B2
ΔG (kJ/mol)	0.34	0	8.77
Pop. (%)	46	53	1

Fig. S4. The different conformations calculated for bipyridine **7** in ethanol

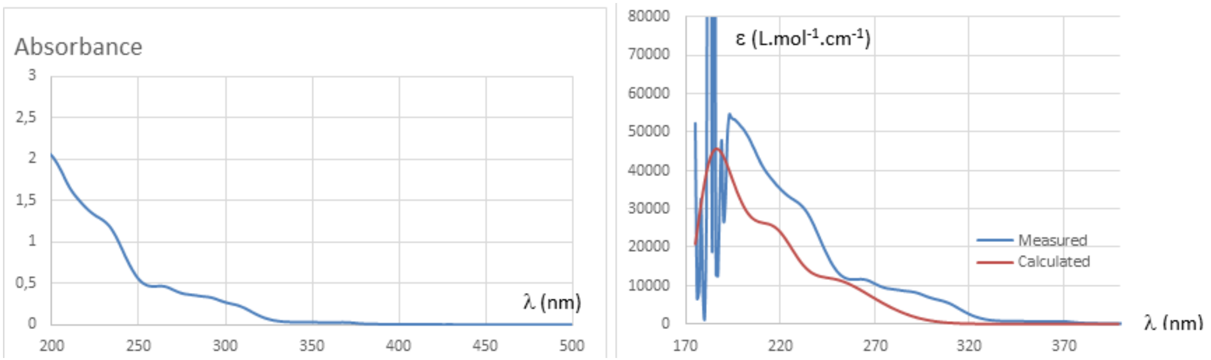

Fig. S5. Comparison of measured and calculated UV spectra for **1** (in ethanol)

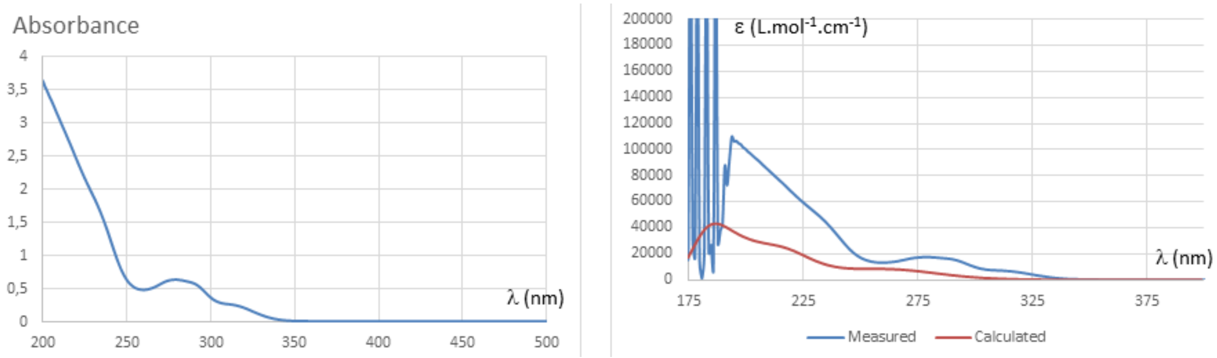

Fig. S6. Comparison of measured and calculated UV spectra for **2** (in ethanol)

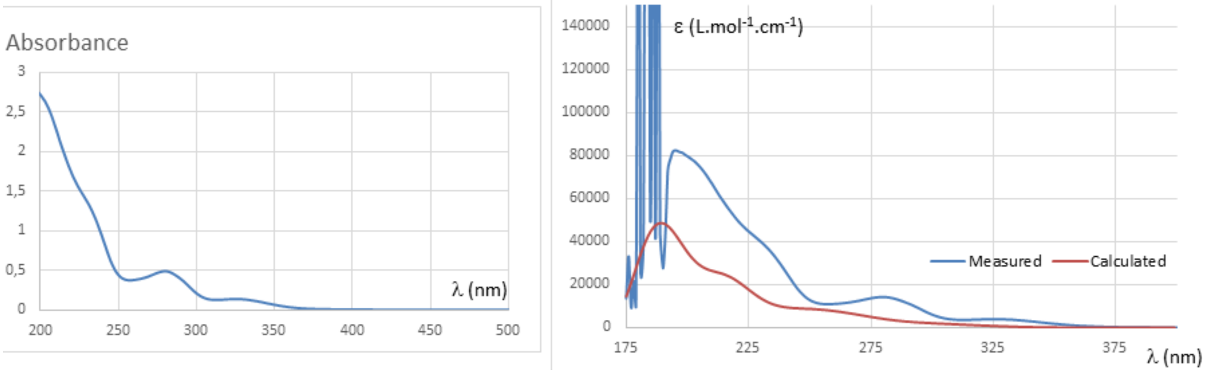

Fig. S7. Comparison of measured and calculated UV spectra for **6** (in ethanol)

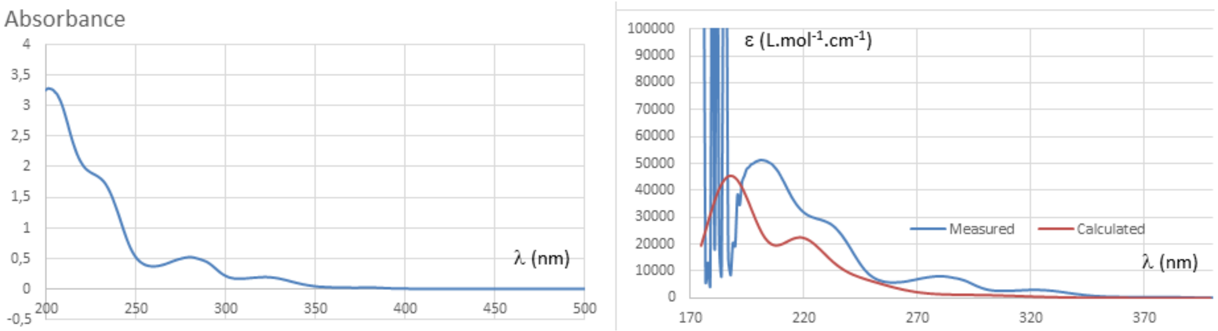

Fig. S8. Comparison of measured and calculated UV spectra for **7** (in ethanol)

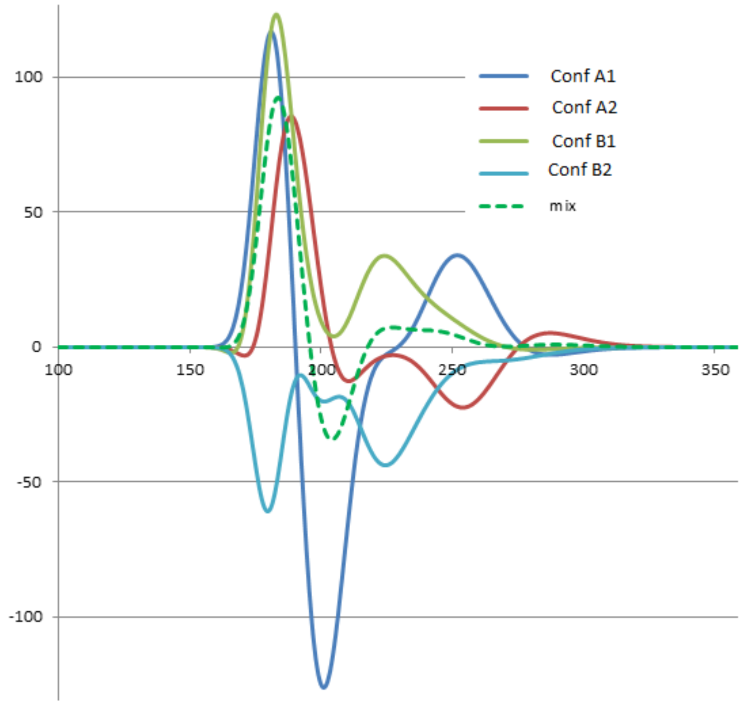

Fig. S9. Calculated ECD spectra for each conformer of bipyridine **(*M*)-1**

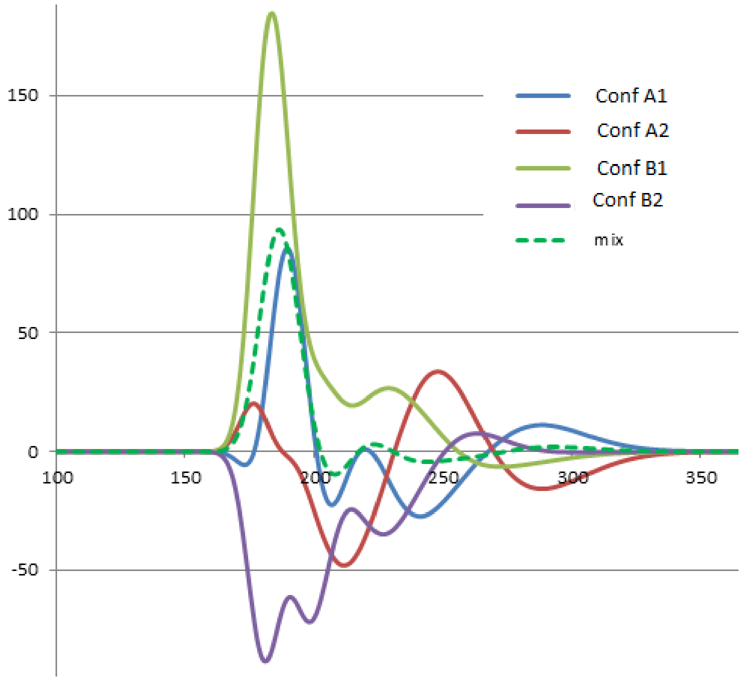

Fig. S10. Calculated ECD spectra for each conformer of bipyridine **(*M*)-2**

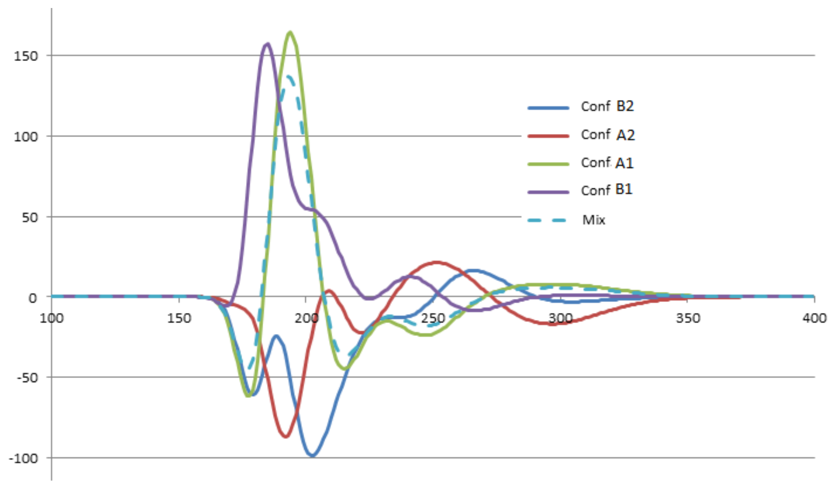

Fig. S11. Calculated ECD spectra for each conformer of bipyridine **(*M*)-6**

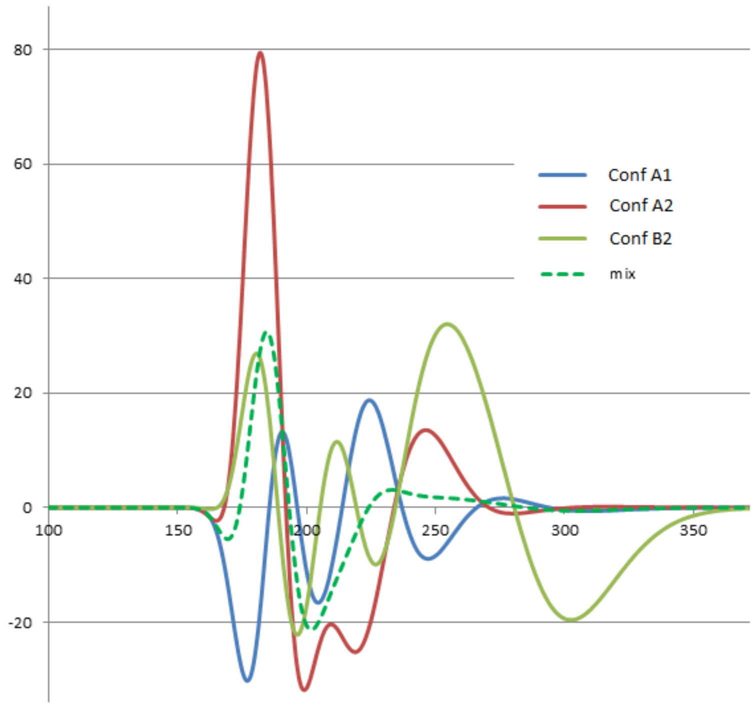

Fig. S12. Calculated ECD spectra for each conformer of bipyridine **(*M*)-7**

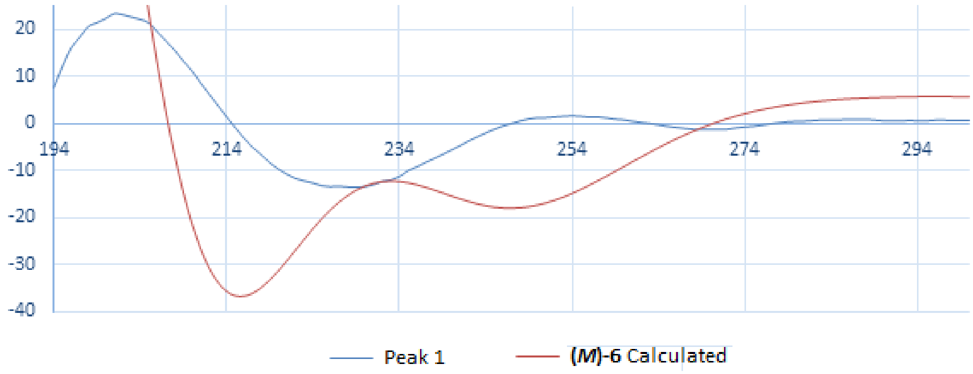

Fig. S13. Comparison of measured and calculated ECD spectra for (*M*)-**6**

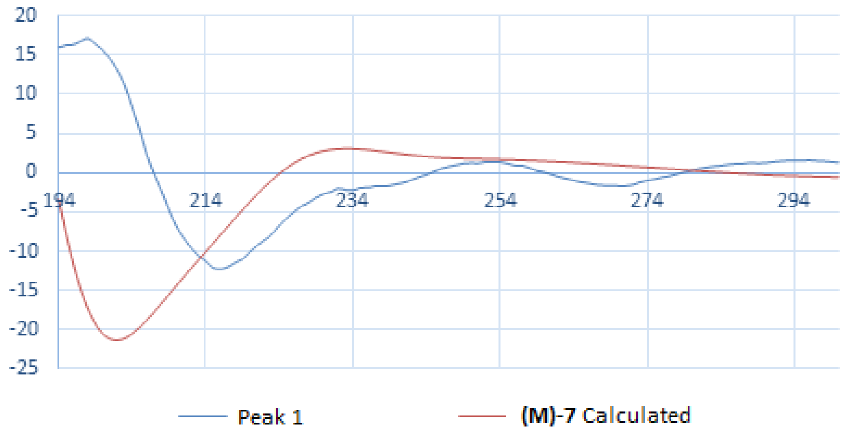

Fig. S14. Comparison of measured and calculated ECD spectra for (*M*)-**7**

II. X-Ray diffraction analysis

The crystals were placed in oil, and a single crystal was selected, mounted on a glass fibre and placed in a low-temperature N_2 stream.

X-ray diffraction data collection was carried out on a Bruker PHOTON III DUO CPAD diffractometer equipped with an Oxford Cryosystem liquid N_2 device, using Mo-Kα radiation (λ = 0.71073 Å). The crystal-detector distance was 37mm. The cell parameters were determined (APEX3 software) [1] from reflections taken from 1 set of 180 frames at 1s exposure. The structure was solved using the program SHELXT-2014 [2]. The refinement and all further calculations were carried out using SHELXL-2014 [3]. The H-atoms were included in calculated positions and treated as riding atoms using SHELXL default parameters. The non-H atoms were refined anisotropically, using weighted full-matrix least-squares on F^2. A semi-empirical absorption correction was applied using SADABS in APEX3 [1]; transmission factors: T_{min}/T_{max} = 0.5542/0.7463.

[1] "M86-EXX229V1 APEX3 User Manual", Bruker AXS Inc., Madison, USA, 2016.

[2] G. M. Sheldrick, *Acta Cryst.* **2015**, A71, 3-8.

[3] G. M. Sheldrick, *Acta Cryst.* **2015**, C71, 3-8.

II.1. Crystal data

Crystal data for **(*P*)-6**

$C_{16}H_8Br_2Cl_2N_2Se$, M = 537.92, monoclinic, *a* = 9.5538(4) Å, *b* = 13.4359(6) Å, *c* = 13.4081(6) Å, β=93.065(2)°, V = 1718.65(13) Å^3, *T* = 120(2) K, space group $P2_1$, Z = 4, μ (Mo Kα) = 7.144 mm^{-1}, 67225 reflections measured, 10077 independent reflections (R_{int} = 0.1084). The final R_1 value were 0.0423 (I > 2σ(I)) and 0.0675 (all data). The final $wR(F^2)$ values were 0.0617 (I > 2σ(I)) and 0.0710 (all data). The goodness of fit on F^2 was 1.053. Flack parameter was 0.010(6) and the absolute configuration of the two independent molecules is (*P*). CCDC no. 1963860.

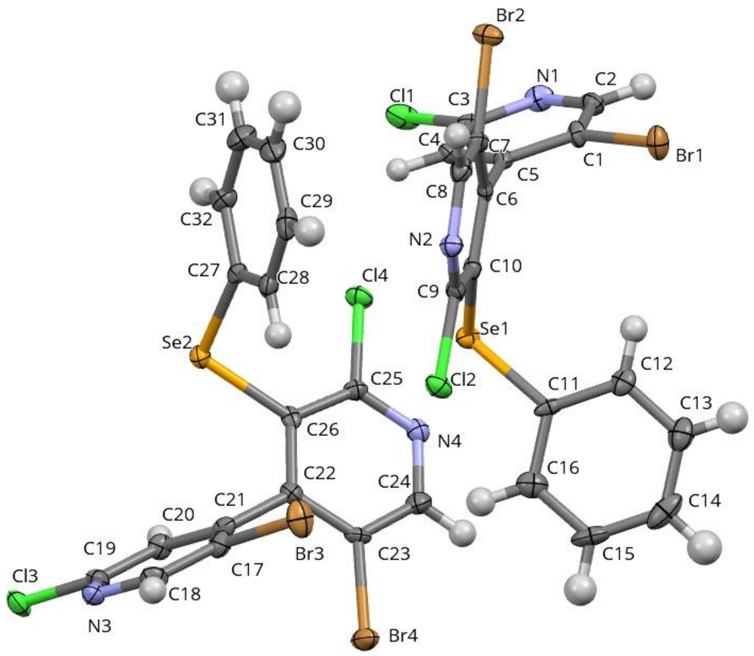

Figure S15. ORTEP plot of **(*P*)-6** with ellipsoids drawn at the 50% probability level.

Table S1. Crystal data and structure refinement for **2**.

Empirical formula	C16 H3 Br2 Cl2 F5 N2 Se
Formula weight	627.88
Temperature	120(2) K
Wavelength	0.71073 A
Crystal system, space group	Monoclinic, $P\ 2_1/c$
Unit cell dimensions	a = 10.9949(5) Å alpha = 90°.<br>b = 13.7036(6) Å beta = 94.897(2) °.<br>c = 12.1716(6) Å gamma = 90°.
Volume	1827.20(15) $Å^3$
Z, Calculated density	4, 2.282 Mg/m^3
Absorption coefficient	6.775 mm^{-1}
F(000)	1184
Crystal size	0.22 x 0.18 x 0.15 mm
Theta range for data collection	2.243 to 32.133 °
Limiting indices	-16<=h<=16, -20<=k<=20, -18<=l<=18
Reflections collected / unique	74843 / 6383 [R(int) = 0.0467]
Completeness to theta = 25.242	99.6 %
Absorption correction	Semi-empirical from equivalents
Max. and min. transmission	0.7463 and 0.5542
Refinement method	Full-matrix least-squares on F^2
Data / restraints / parameters	6383 / 0 / 253
Goodness-of-fit on F^2	1.117
Final R indices [I>2sigma(I)]	R1 = 0.0221, wR2 = 0.0552
R indices (all data)	R1 = 0.0240, wR2 = 0.0560
Extinction coefficient	n/a
Largest diff. peak and hole	1.021 and -0.694 e. $Å^{-3}$

Table S2. Atomic coordinates (x 10^4) and equivalent isotropic displacement parameters (A^2 x 10^3) for **2**. U(eq) is defined as one third of the trace of the orthogonalized Uij tensor.

	x	y	z	U(eq)
C(1)	2225(2)	2830(1)	4457(1)	14(1)
C(2)	1112(2)	3134(1)	4794(1)	17(1)
C(3)	966(2)	1770(1)	5828(1)	16(1)
C(4)	2074(2)	1395(1)	5545(1)	15(1)
C(5)	2719(1)	1940(1)	4820(1)	14(1)
C(6)	3847(1)	1520(1)	4408(1)	13(1)
C(7)	3771(2)	1033(1)	3394(1)	15(1)
C(8)	4797(2)	584(1)	3039(1)	17(1)
C(9)	5957(2)	1069(1)	4589(1)	16(1)
C(10)	4981(2)	1553(1)	5025(1)	13(1)
C(11)	6520(2)	2911(1)	6397(1)	16(1)
C(12)	7427(2)	2848(1)	7261(1)	19(1)
C(13)	8493(2)	3389(1)	7276(2)	19(1)
C(14)	8671(2)	3993(1)	6392(2)	18(1)
C(15)	7792(2)	4058(1)	5519(2)	19(1)
C(16)	6718(2)	3538(1)	5537(1)	18(1)
N(1)	479(1)	2602(1)	5473(1)	18(1)
N(2)	5878(1)	599(1)	3635(1)	17(1)
F(1)	7289(1)	2250(1)	8110(1)	31(1)
F(2)	9348(1)	3317(1)	8112(1)	31(1)
F(3)	9695(1)	4509(1)	6381(1)	23(1)
F(4)	7985(1)	4636(1)	4669(1)	31(1)
F(5)	5869(1)	3643(1)	4690(1)	27(1)
Cl(1)	138(1)	1093(1)	6712(1)	25(1)
Cl(2)	7387(1)	1037(1)	5302(1)	21(1)

Se(1)	5057(1)	2179(1)	6439(1)	19(1)
Br(1)	3048(1)	3637(1)	3509(1)	19(1)
Br(2)	2291(1)	965(1)	2502(1)	22(1)

Table S3. Bond lengths [Å] and angles [°] for **2**.

C(1)-C(2)	1.388(2)
C(1)-C(5)	1.392(2)
C(1)-Br(1)	1.8843(16)
C(2)-N(1)	1.340(2)
C(2)-H(2)	0.9500
C(3)-N(1)	1.316(2)
C(3)-C(4)	1.392(2)
C(3)-Cl(1)	1.7369(17)
C(4)-C(5)	1.395(2)
C(4)-H(4)	0.9500
C(5)-C(6)	1.492(2)
C(6)-C(7)	1.399(2)
C(6)-C(10)	1.400(2)
C(7)-C(8)	1.386(2)
C(7)-Br(2)	1.8794(16)
C(8)-N(2)	1.338(2)
C(8)-H(8)	0.9500
C(9)-N(2)	1.325(2)
C(9)-C(10)	1.404(2)
C(9)-Cl(2)	1.7298(17)
C(10)-Se(1)	1.9190(16)
C(11)-C(16)	1.385(2)
C(11)-C(12)	1.389(2)

C(11)-Se(1)	1.9005(17)
C(12)-F(1)	1.338(2)
C(12)-C(13)	1.386(2)
C(13)-F(2)	1.329(2)
C(13)-C(14)	1.384(3)
C(14)-F(3)	1.3316(19)
C(14)-C(15)	1.377(3)
C(15)-F(4)	1.334(2)
C(15)-C(16)	1.381(2)
C(16)-F(5)	1.338(2)
C(2)-C(1)-C(5)	119.94(15)
C(2)-C(1)-Br(1)	119.12(13)
C(5)-C(1)-Br(1)	120.94(12)
N(1)-C(2)-C(1)	122.44(16)
N(1)-C(2)-H(2)	118.8
C(1)-C(2)-H(2)	118.8
N(1)-C(3)-C(4)	125.34(16)
N(1)-C(3)-Cl(1)	116.48(13)
C(4)-C(3)-Cl(1)	118.18(13)
C(3)-C(4)-C(5)	117.49(15)
C(3)-C(4)-H(4)	121.3
C(5)-C(4)-H(4)	121.3
C(1)-C(5)-C(4)	117.67(14)
C(1)-C(5)-C(6)	122.98(14)
C(4)-C(5)-C(6)	119.21(14)
C(7)-C(6)-C(10)	118.17(14)
C(7)-C(6)-C(5)	119.42(14)
C(10)-C(6)-C(5)	122.35(14)
C(8)-C(7)-C(6)	119.84(15)

C(8)-C(7)-Br(2)	119.02(12)
C(6)-C(7)-Br(2)	121.13(12)
N(2)-C(8)-C(7)	122.20(15)
N(2)-C(8)-H(8)	118.9
C(7)-C(8)-H(8)	118.9
N(2)-C(9)-C(10)	124.44(15)
N(2)-C(9)-Cl(2)	114.63(12)
C(10)-C(9)-Cl(2)	120.93(13)
C(6)-C(10)-C(9)	117.18(14)
C(6)-C(10)-Se(1)	117.65(11)
C(9)-C(10)-Se(1)	125.03(12)
C(16)-C(11)-C(12)	117.49(16)
C(16)-C(11)-Se(1)	122.40(13)
C(12)-C(11)-Se(1)	120.10(13)
F(1)-C(12)-C(13)	118.01(16)
F(1)-C(12)-C(11)	120.07(16)
C(13)-C(12)-C(11)	121.92(16)
F(2)-C(13)-C(14)	120.19(16)
F(2)-C(13)-C(12)	120.81(17)
C(14)-C(13)-C(12)	118.98(16)
F(3)-C(14)-C(15)	119.73(16)
F(3)-C(14)-C(13)	120.08(16)
C(15)-C(14)-C(13)	120.19(16)
F(4)-C(15)-C(14)	119.31(16)
F(4)-C(15)-C(16)	120.77(16)
C(14)-C(15)-C(16)	119.92(16)
F(5)-C(16)-C(15)	118.23(16)
F(5)-C(16)-C(11)	120.33(16)
C(15)-C(16)-C(11)	121.43(16)

C(3)-N(1)-C(2)	117.10(15)
C(9)-N(2)-C(8)	118.15(14)
C(11)-Se(1)-C(10)	100.58(7)

Table S4. Anisotropic displacement parameters ($Å^2$ x 10^3) for **2**.

The anisotropic displacement factor exponent takes the form:

$-2 \pi^2 [h^2 a^{*2} U11 + ... + 2 h k a^* b^* U12]$

	U11	U22	U33	U23	U13	U12
C(1)	13(1)	16(1)	14(1)	0(1)	2(1)	0(1)
C(2)	15(1)	17(1)	20(1)	-2(1)	2(1)	3(1)
C(3)	12(1)	22(1)	16(1)	0(1)	2(1)	-2(1)
C(4)	13(1)	16(1)	17(1)	-1(1)	2(1)	1(1)
C(5)	11(1)	16(1)	13(1)	-2(1)	1(1)	2(1)
C(6)	13(1)	13(1)	13(1)	1(1)	3(1)	2(1)
C(7)	14(1)	16(1)	15(1)	0(1)	1(1)	2(1)
C(8)	19(1)	16(1)	16(1)	-3(1)	4(1)	3(1)
C(9)	11(1)	15(1)	21(1)	2(1)	2(1)	1(1)
C(10)	13(1)	14(1)	14(1)	-1(1)	2(1)	-1(1)
C(11)	15(1)	18(1)	16(1)	-3(1)	2(1)	0(1)
C(12)	20(1)	22(1)	16(1)	1(1)	1(1)	-2(1)
C(13)	16(1)	22(1)	20(1)	-2(1)	-1(1)	1(1)
C(14)	14(1)	16(1)	23(1)	-5(1)	4(1)	-2(1)
C(15)	22(1)	16(1)	19(1)	1(1)	3(1)	-2(1)

C(16)	18(1)	18(1)	16(1)	-2(1)	-1(1)	1(1)
N(1)	12(1)	21(1)	20(1)	-2(1)	2(1)	2(1)
N(2)	16(1)	16(1)	20(1)	-2(1)	5(1)	3(1)
F(1)	33(1)	39(1)	20(1)	12(1)	-3(1)	-11(1)
F(2)	23(1)	40(1)	28(1)	5(1)	-10(1)	-6(1)
F(3)	17(1)	20(1)	31(1)	-3(1)	4(1)	-6(1)
F(4)	40(1)	28(1)	24(1)	8(1)	2(1)	-11(1)
F(5)	26(1)	31(1)	22(1)	4(1)	-8(1)	-3(1)
Cl(1)	15(1)	32(1)	27(1)	8(1)	6(1)	-1(1)
Cl(2)	12(1)	21(1)	31(1)	-5(1)	-1(1)	3(1)
Se(1)	15(1)	26(1)	15(1)	-5(1)	4(1)	-4(1)
Br(1)	19(1)	19(1)	21(1)	4(1)	6(1)	3(1)
Br(2)	19(1)	29(1)	17(1)	-6(1)	-5(1)	4(1)

Table S5. Torsion angles [°] for **2**.

C(5)-C(1)-C(2)-N(1)	0.5(3)
Br(1)-C(1)-C(2)-N(1)	-179.53(13)
N(1)-C(3)-C(4)-C(5)	-0.5(3)
Cl(1)-C(3)-C(4)-C(5)	178.96(12)
C(2)-C(1)-C(5)-C(4)	-1.8(2)
Br(1)-C(1)-C(5)-C(4)	178.22(12)
C(2)-C(1)-C(5)-C(6)	173.80(15)
Br(1)-C(1)-C(5)-C(6)	-6.2(2)
C(3)-C(4)-C(5)-C(1)	1.8(2)
C(3)-C(4)-C(5)-C(6)	-174.00(15)
C(1)-C(5)-C(6)-C(7)	-80.4(2)
C(4)-C(5)-C(6)-C(7)	95.19(19)
C(1)-C(5)-C(6)-C(10)	102.69(19)

C(4)-C(5)-C(6)-C(10)	-81.7(2)
C(10)-C(6)-C(7)-C(8)	1.4(2)
C(5)-C(6)-C(7)-C(8)	-175.65(15)
C(10)-C(6)-C(7)-Br(2)	-179.23(12)
C(5)-C(6)-C(7)-Br(2)	3.7(2)
C(6)-C(7)-C(8)-N(2)	-0.2(3)
Br(2)-C(7)-C(8)-N(2)	-179.62(13)
C(7)-C(6)-C(10)-C(9)	-1.7(2)
C(5)-C(6)-C(10)-C(9)	175.27(15)
C(7)-C(6)-C(10)-Se(1)	-177.69(12)
C(5)-C(6)-C(10)-Se(1)	-0.7(2)
N(2)-C(9)-C(10)-C(6)	0.9(3)
Cl(2)-C(9)-C(10)-C(6)	-178.28(12)
N(2)-C(9)-C(10)-Se(1)	176.60(13)
Cl(2)-C(9)-C(10)-Se(1)	-2.6(2)
C(16)-C(11)-C(12)-F(1)	-179.59(16)
Se(1)-C(11)-C(12)-F(1)	2.0(2)
C(16)-C(11)-C(12)-C(13)	0.2(3)
Se(1)-C(11)-C(12)-C(13)	-178.24(14)
F(1)-C(12)-C(13)-F(2)	-0.2(3)
C(11)-C(12)-C(13)-F(2)	-179.98(17)
F(1)-C(12)-C(13)-C(14)	178.24(16)
C(11)-C(12)-C(13)-C(14)	-1.5(3)
F(2)-C(13)-C(14)-F(3)	-0.6(3)
C(12)-C(13)-C(14)-F(3)	-179.07(16)
F(2)-C(13)-C(14)-C(15)	179.09(17)
C(12)-C(13)-C(14)-C(15)	0.6(3)
F(3)-C(14)-C(15)-F(4)	0.4(3)
C(13)-C(14)-C(15)-F(4)	-179.35(16)

F(3)-C(14)-C(15)-C(16)	-178.70(16)
C(13)-C(14)-C(15)-C(16)	1.6(3)
F(4)-C(15)-C(16)-F(5)	-1.1(3)
C(14)-C(15)-C(16)-F(5)	177.91(16)
F(4)-C(15)-C(16)-C(11)	177.95(16)
C(14)-C(15)-C(16)-C(11)	-3.0(3)
C(12)-C(11)-C(16)-F(5)	-178.84(16)
Se(1)-C(11)-C(16)-F(5)	-0.5(2)
C(12)-C(11)-C(16)-C(15)	2.1(3)
Se(1)-C(11)-C(16)-C(15)	-179.51(13)
C(4)-C(3)-N(1)-C(2)	-0.8(3)
Cl(1)-C(3)-N(1)-C(2)	179.70(13)
C(1)-C(2)-N(1)-C(3)	0.8(3)
C(10)-C(9)-N(2)-C(8)	0.2(3)
Cl(2)-C(9)-N(2)-C(8)	179.49(13)
C(7)-C(8)-N(2)-C(9)	-0.6(3)

II.1 Intermolecular interactions

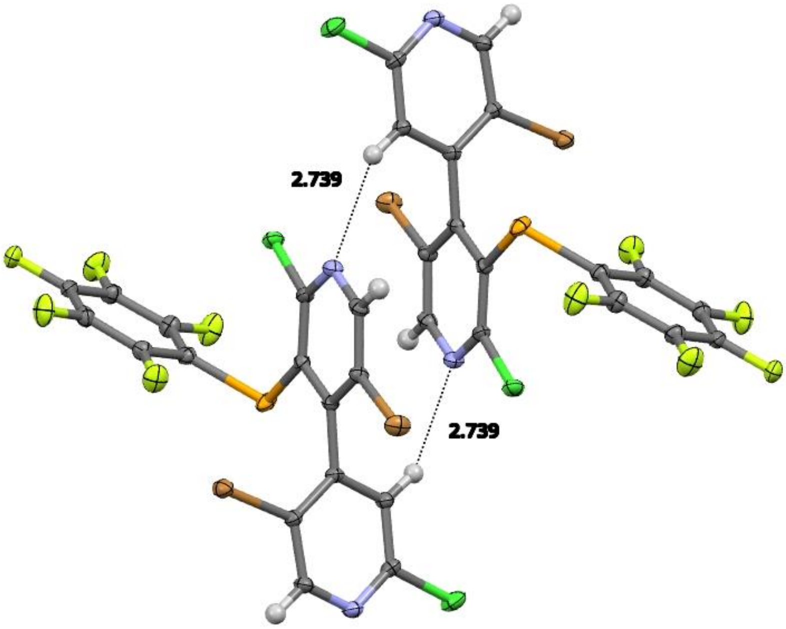

Figure S16. Hydrogen bonds

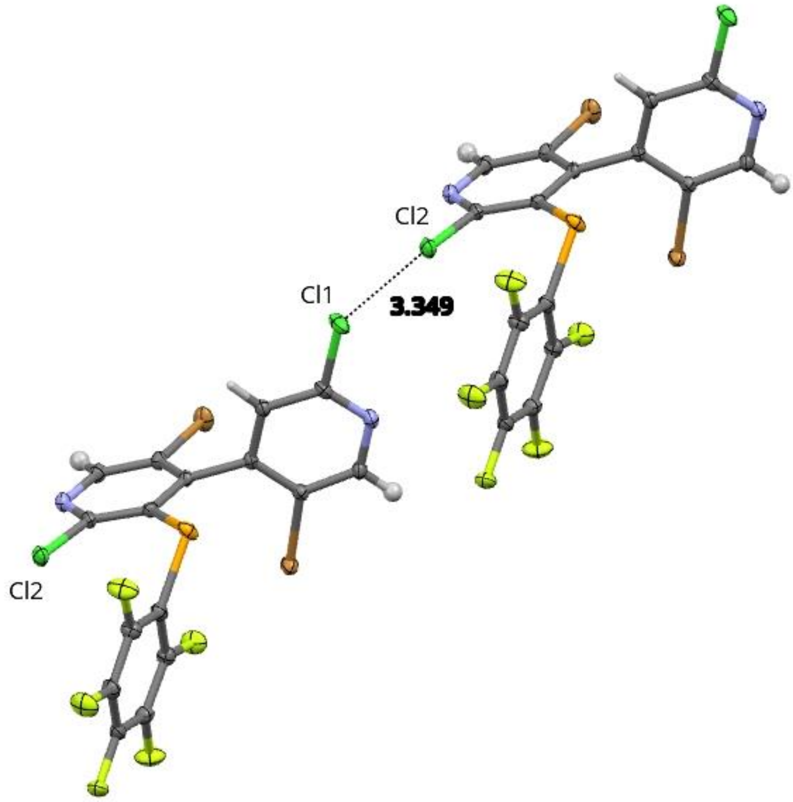

Figure S17. Type II halogen···halogen bond

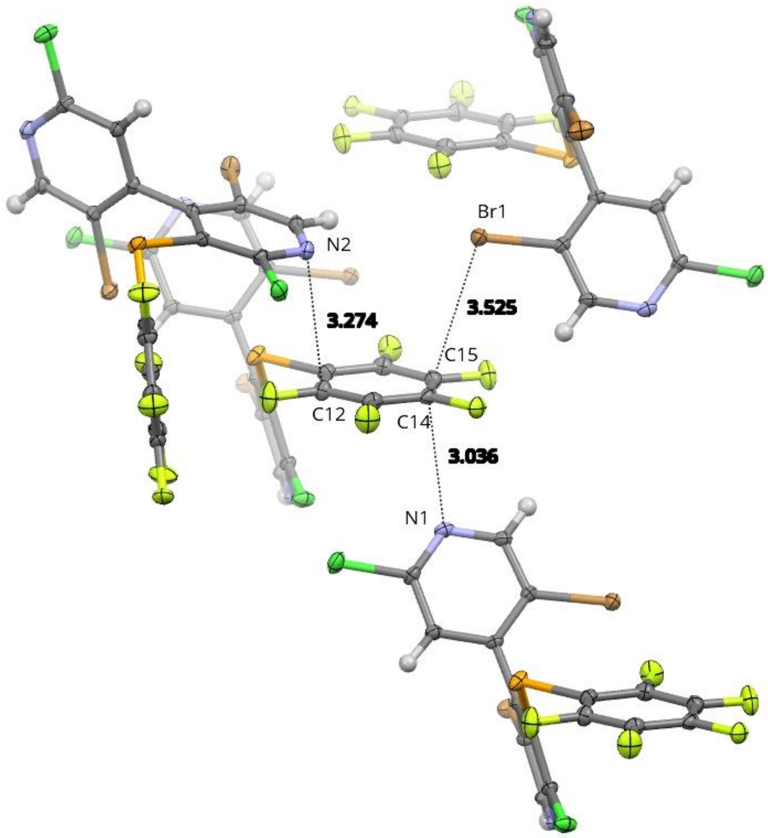

Figure S18. Interactions of C_6F_5 π-hole

III. DFT details

DFT calculations were performed with Gaussian09 [1] software employing Density Function Theory. Relaxed Potential Energy Surfaces were first explored by scanning the torsion angle about the C_{py}-S,Se bond at the CAM-B3LYP-D3/6-311G(d,p) level of theory. The resulting conformers were then fully optimized employing the same functional but the larger basis set 6-311+G(d,p); the effect of solvent (EtOH) were taken into account through a PCM model and frequency calculations were then performed to check that true energy minima were obtained (no imaginary frequency). Time Dependent DFT calculations were performed on the optimized structures with the same functional and basis set in order to simulate the ECD spectra; 48 excited states were calculated, and the resulting spectra were obtained for each conformer using peak half-width at half height of 0.3eV. The final spectra, representative of each investigated compounds, were calculated as Boltzmann weighted average of the individual conformers, taken into account their relative energy stabilities. (see Fig. S3-S8).

[1] Gaussian 09, Revision D.01, M. J. Frisch, G. W. Trucks, H. B. Schlegel, G. E. Scuseria, M. A. Robb, J. R. Cheeseman, G. Scalmani, V. Barone, B. Mennucci, G. A. Petersson, H. Nakatsuji, M. Caricato, X. Li, H. P. Hratchian, A. F. Izmaylov, J. Bloino, G. Zheng, J. L. Sonnenberg, M. Hada, M. Ehara, K. Toyota, R. Fukuda, J. Hasegawa, M. Ishida, T. Nakajima, Y. Honda, O. Kitao, H. Nakai, T. Vreven, J. A. Montgomery, Jr., J. E. Peralta, F. Ogliaro, M. Bearpark, J. J. Heyd, E. Brothers, K. N. Kudin, V. N. Staroverov, T. Keith, R. Kobayashi, J. Normand, K. Raghavachari, A. Rendell, J. C. Burant, S. S. Iyengar, J. Tomasi, M. Cossi, N. Rega, J. M. Millam, M. Klene, J. E. Knox, J. B. Cross, V. Bakken, C. Adamo, J. Jaramillo, R. Gomperts, R. E. Stratmann, O. Yazyev, A. J. Austin, R. Cammi, C. Pomelli, J. W. Ochterski, R. L. Martin, K. Morokuma, V. G. Zakrzewski, G. A. Voth, P. Salvador, J. J. Dannenberg, S. Dapprich, A. D. Daniels, O. Farkas, J. B. Foresman, J. V. Ortiz, J. Cioslowski, and D. J. Fox, Gaussian, Inc., Wallingford CT, 2013.

IV. Anion binding experiments

For pipetting Hamilton®-syringes were used. All experiments were conducted at ambient temperature (298.5 K) and in NMR tubes. A 55 mM stock solution of the host in CD_2Cl_2 was prepared. A stock-solution of tetra-*n*-butylammonium chloride (TBACl) (chalcogen bond acceptor/guest) was prepared as 1.5 M solution. For the titration 100 µL of the host were added to the NMR tube and diluted with 400 µL CD_2Cl_2. Then the respective amount of guest solution (1eq. ≡1µl) was added for each data point as shown in Table S1. The ^{19}F NMR spectra were measured. Upon each addition of the guest solution, samples were thoroughly shaken in the NMR tube and then allowed to equilibrate for up to 2 min inside the NMR probe before the spectra were taken. Throughout each titration experiment all parameters of the NMR spectrometer remained constant. Resonances of ortho-, meta- and para-F were followed, allowing several data sets to use in determination of the association constant. Global fitting takes into account all data sets at the same time and improves the quality of the nonlinear curve fitting.

Table S6: Overview of host addition

Equivalents	Added amount (1µl) of the guest solution
1	1
2	1
4	1
5	1
10	5
15	5
20	5
25	5
30	5
40	10
60	20
80	20
100	20
150	50
200	50

For the determination of the binding constants the shift of the aromatic fluorine were observed (Figures S11 and S12). The measured shifts were plotted against the guest-equivalents and the resulting curve was fitted using http://supramolecular.org/. For the calculation of the binding constants (K) a 1:1 binding was assumed.

Table S7: Binding constants Ka

Entry	Host	Guest	Solvent	Ka [M^{-1}]
**1**	Compound **1**	TBACl	DCM-d_2	0.40
**2**	Compound **2**	TBACl	DCM-d_2	38

V. NMR spectra

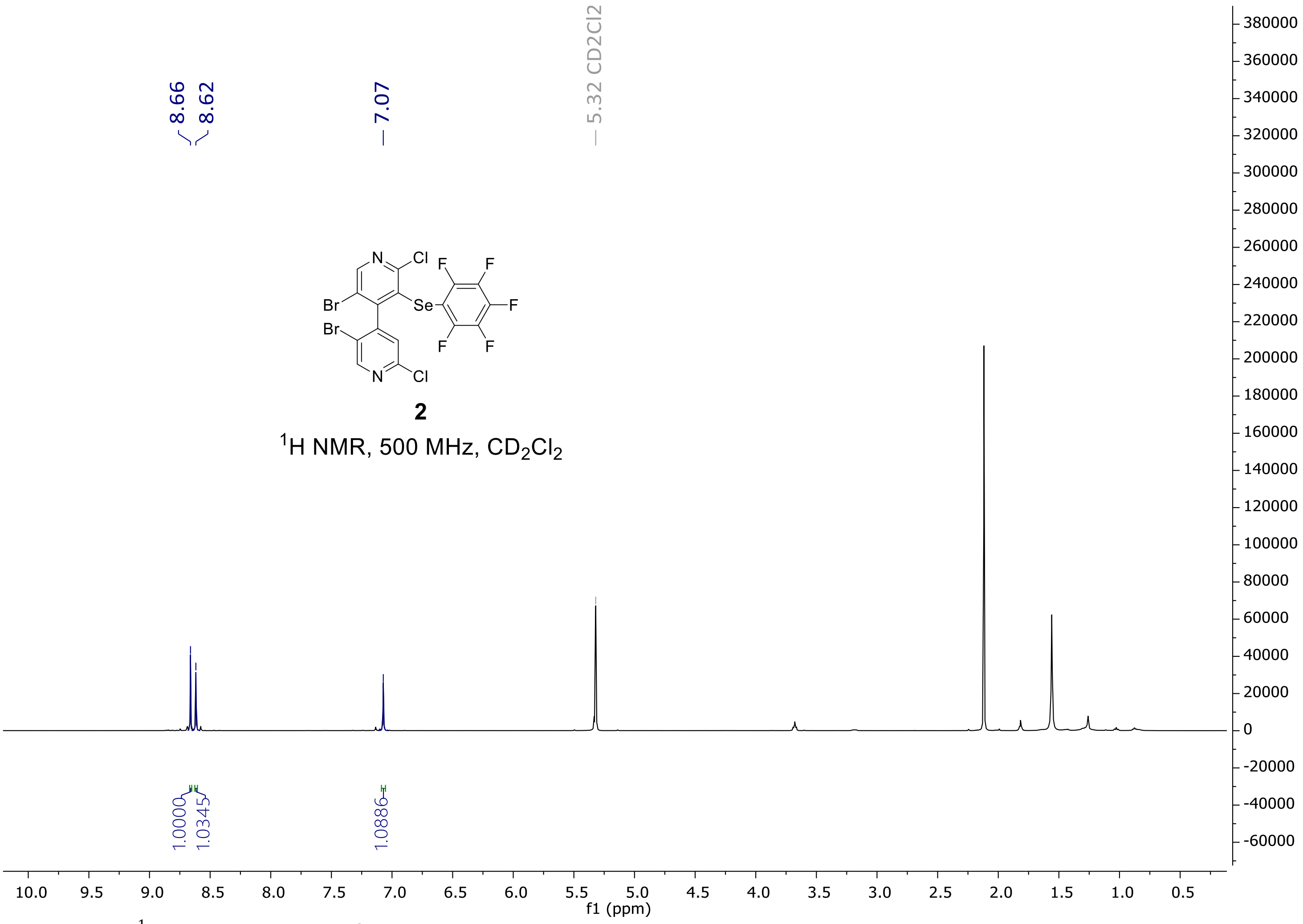

Figure S19. ^{1}H NMR spectrum of **2**

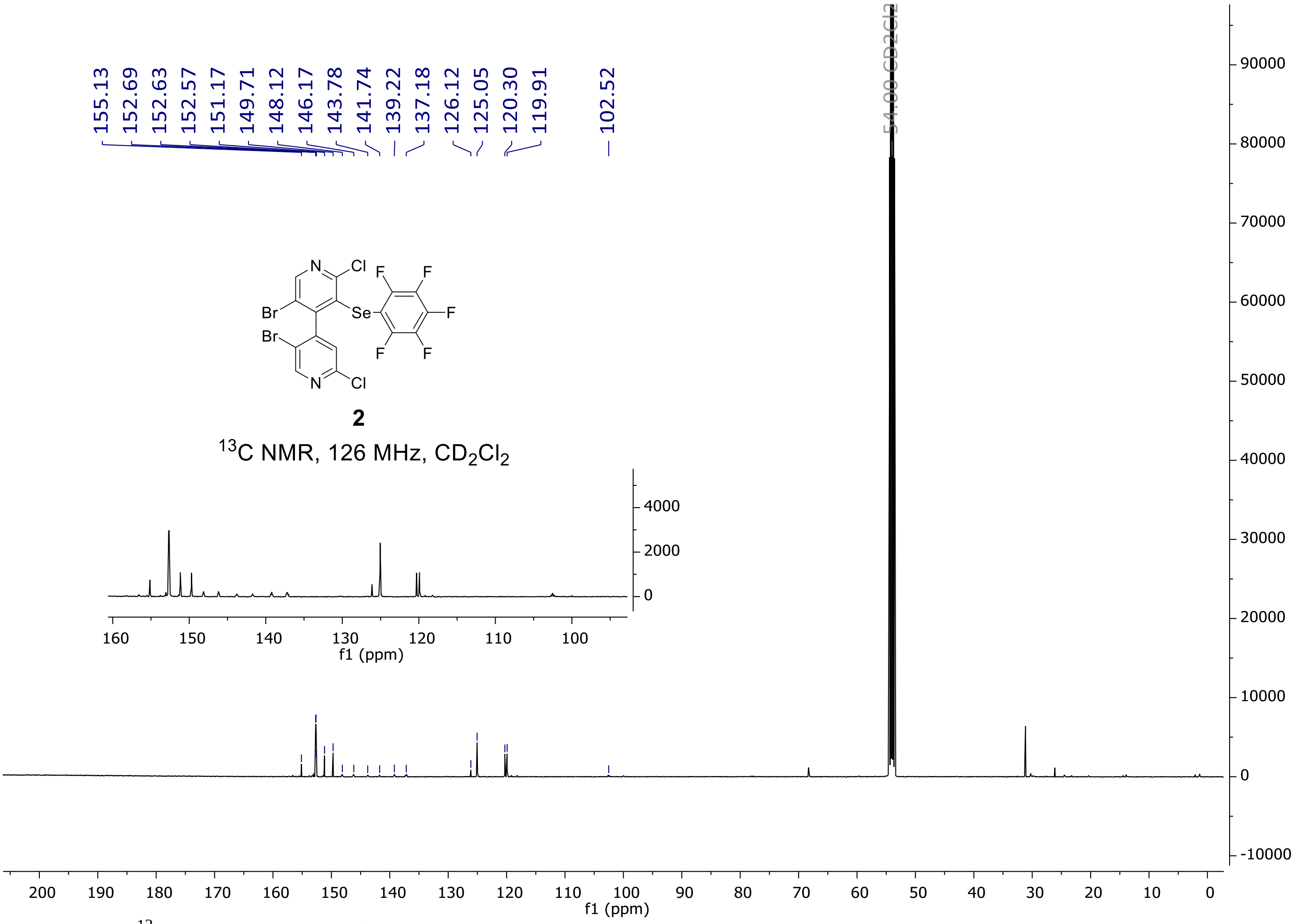

Figure S20. ^{13}C NMR spectrum of **2**

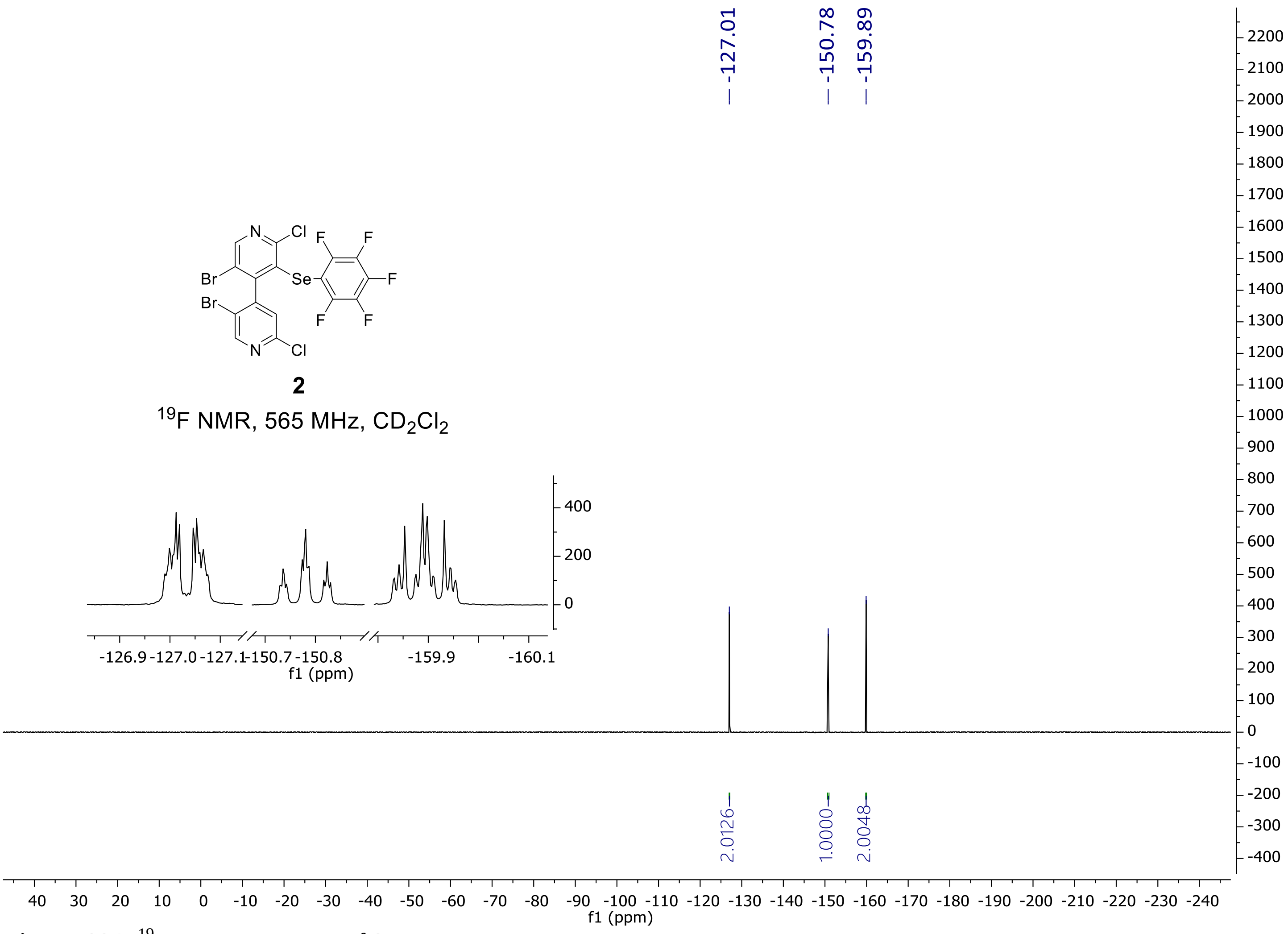

Figure S21. ^{19}F NMR spectrum of **2**

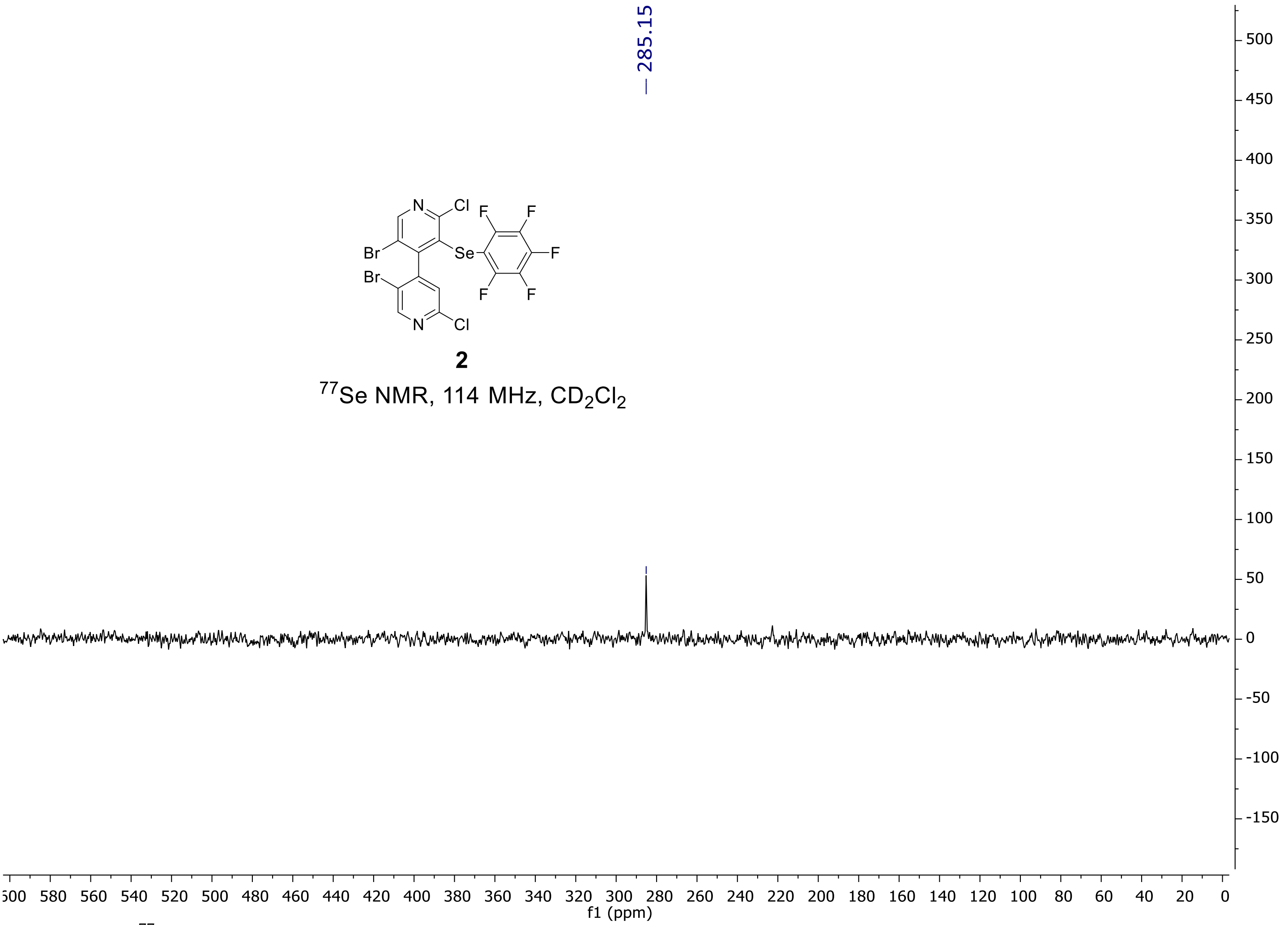

Figure S22. ^{77}Se NMR spectrum of **2**

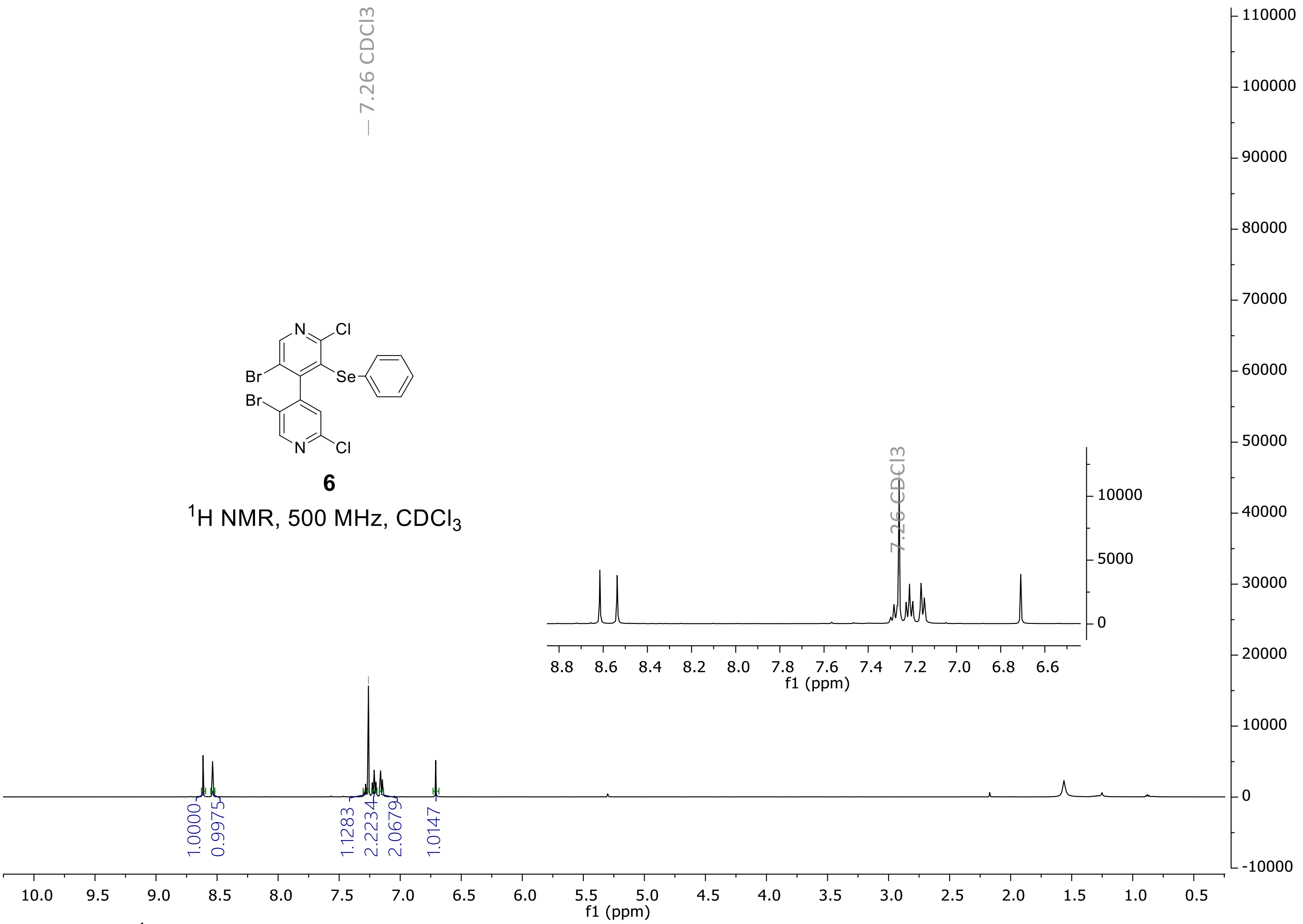

Figure S23. ^{1}H NMR spectrum of **6**

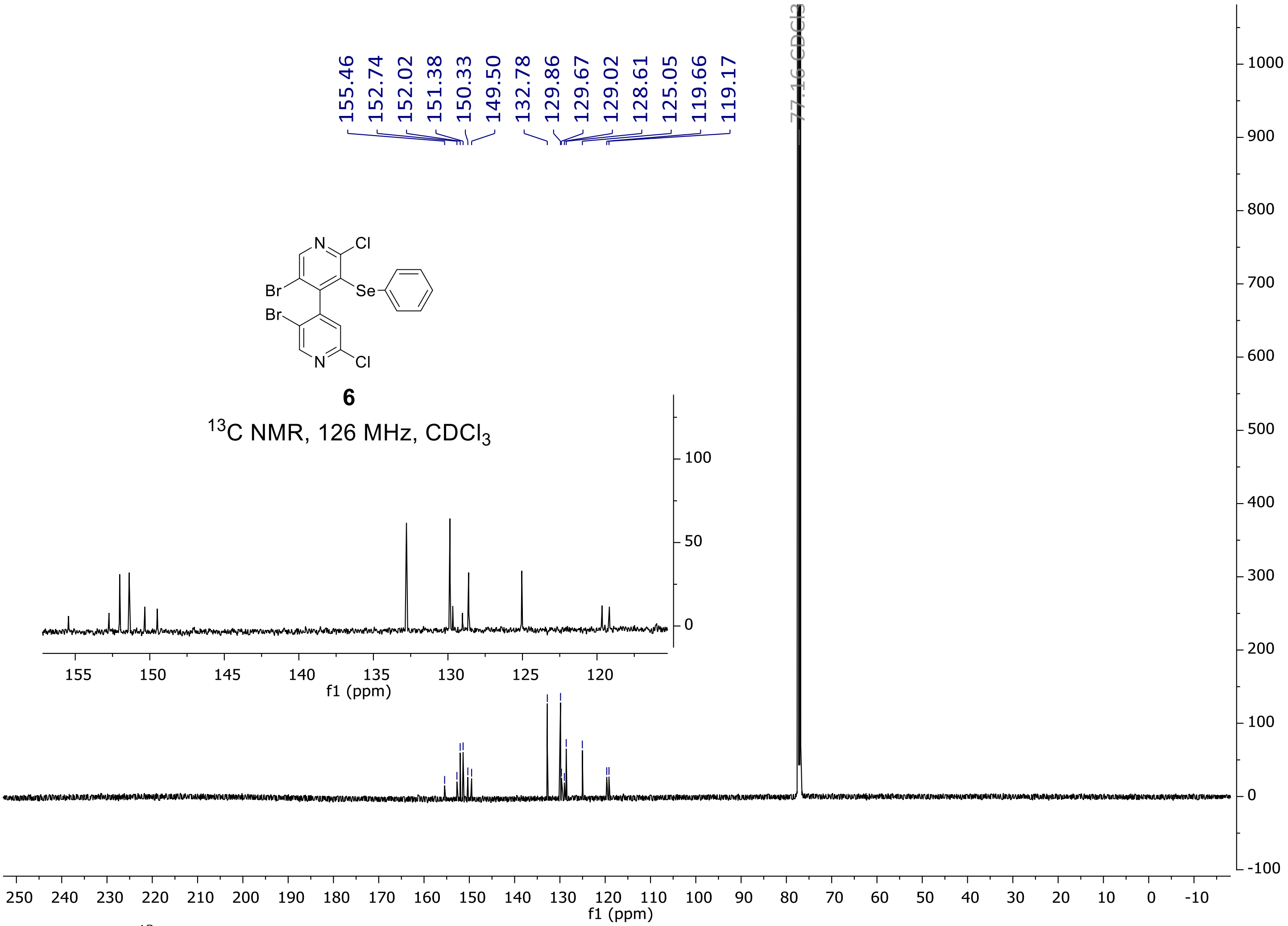

Figure S24. ^{13}C NMR spectrum of **6**

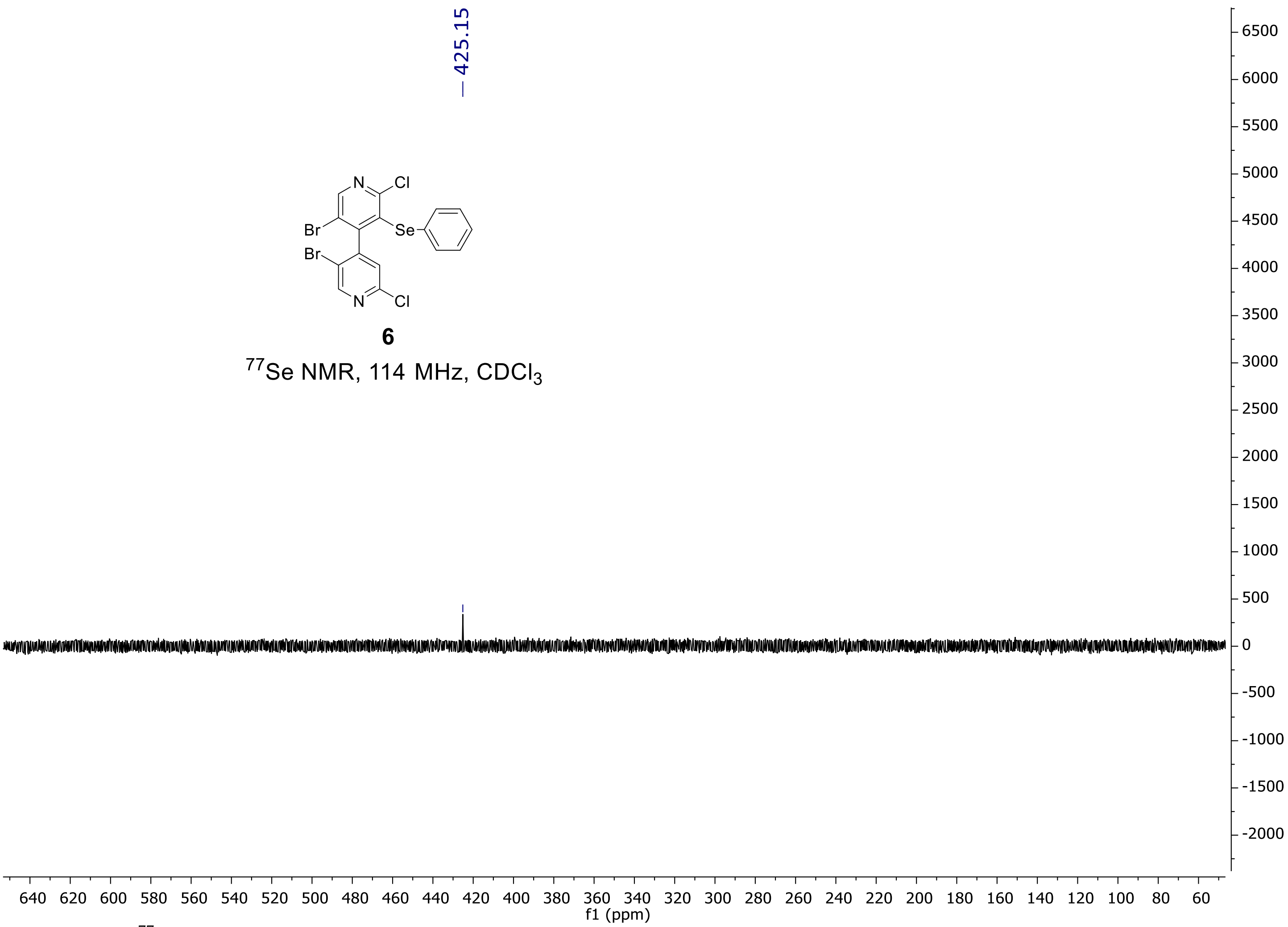

Figure S25. ^{77}Se NMR spectrum of **6**

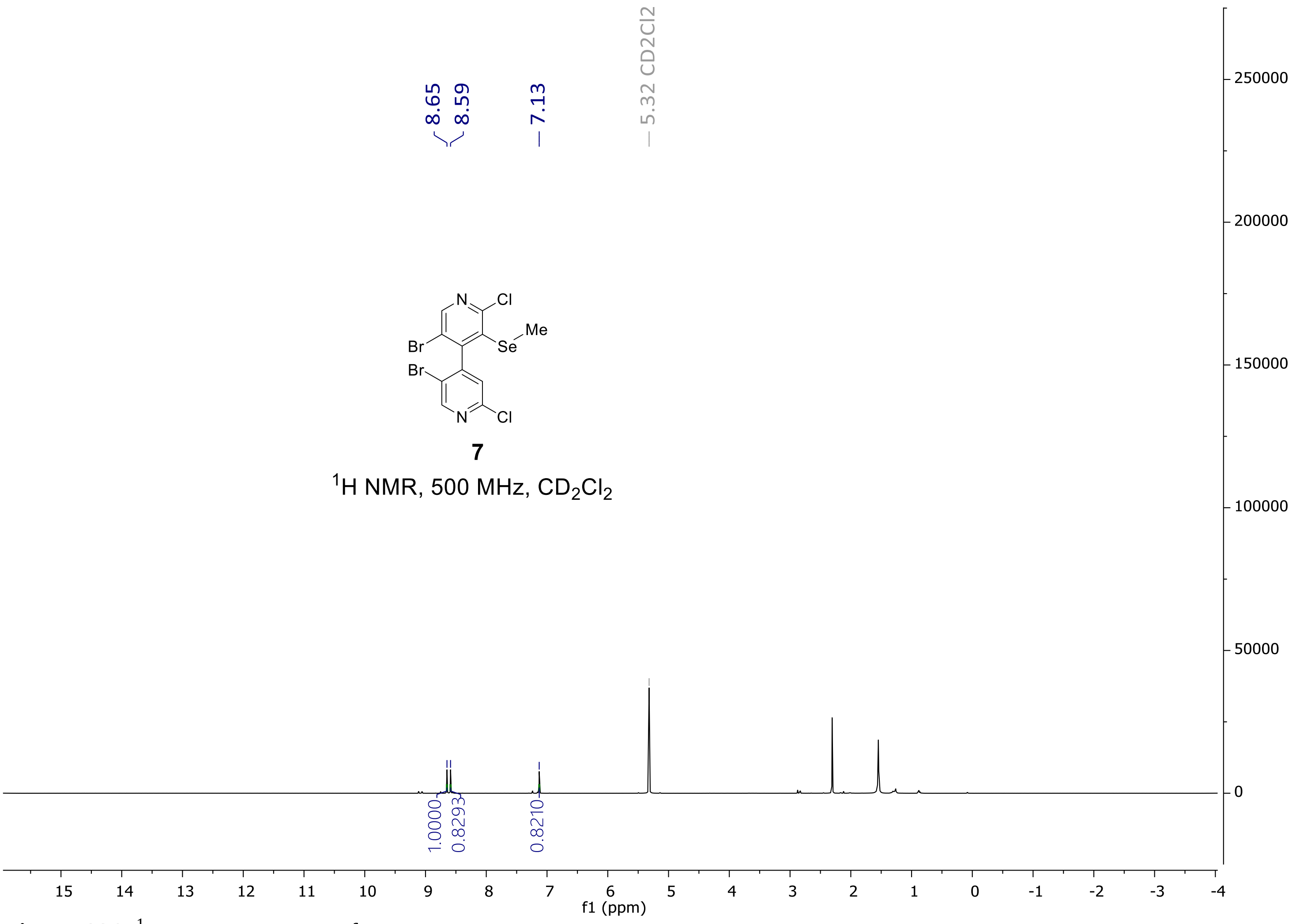

Figure S26. ^{1}H NMR spectrum of **7**

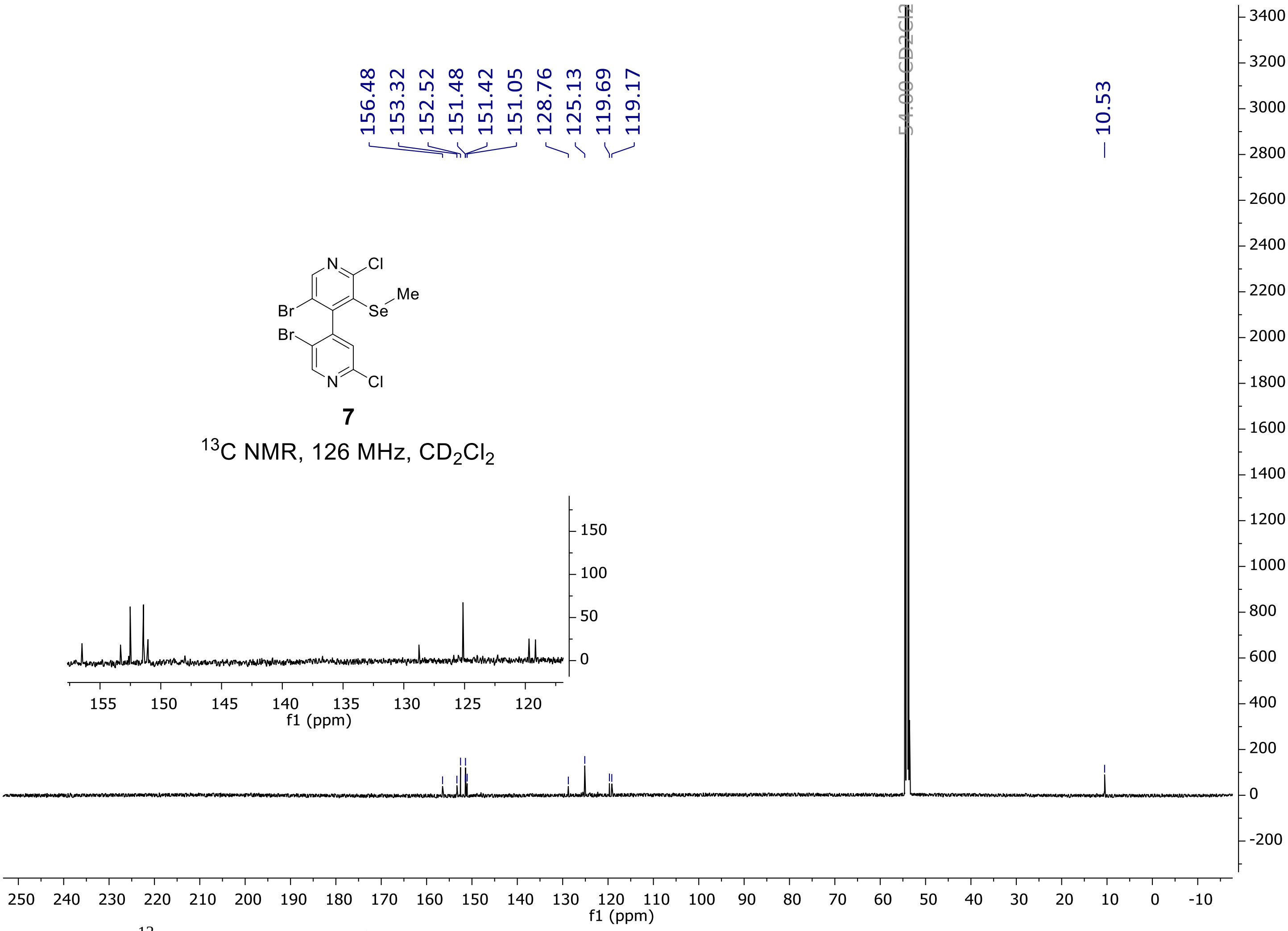

Figure S27. ^{13}C NMR spectrum of **7**

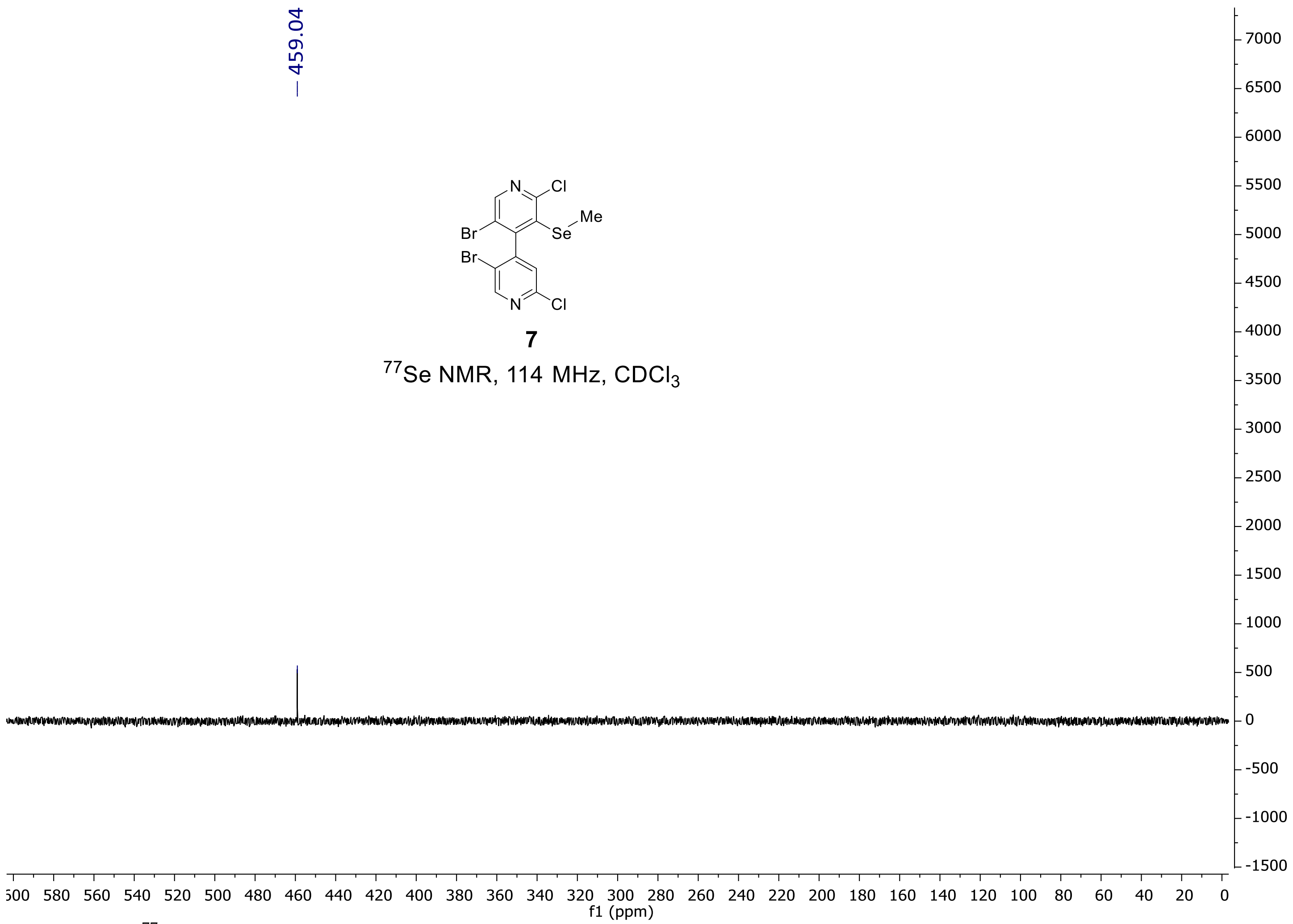

Figure S28. ^{77}Se NMR spectrum of **7**

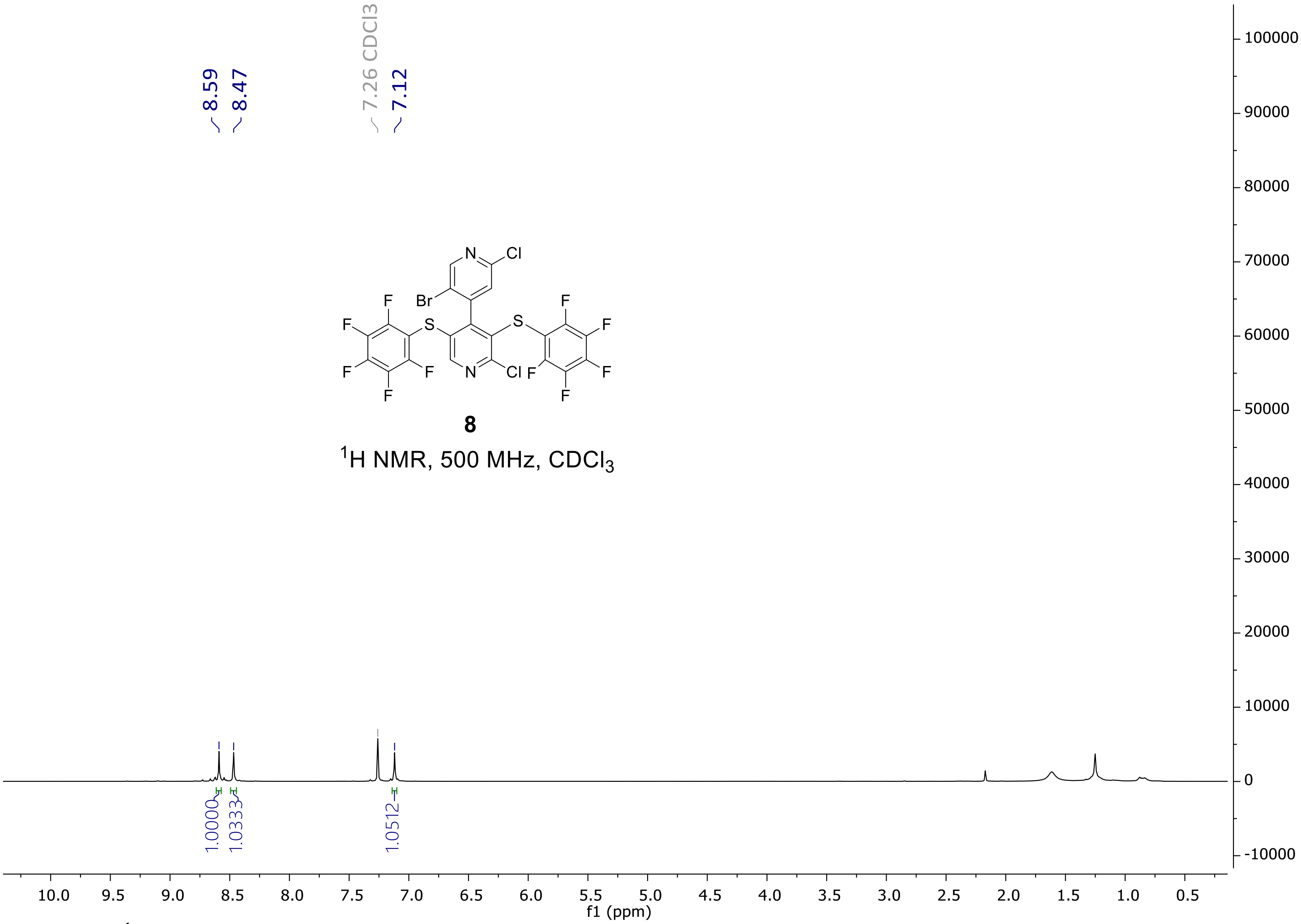

Figure 29. ^{1}H NMR spectrum of **8**

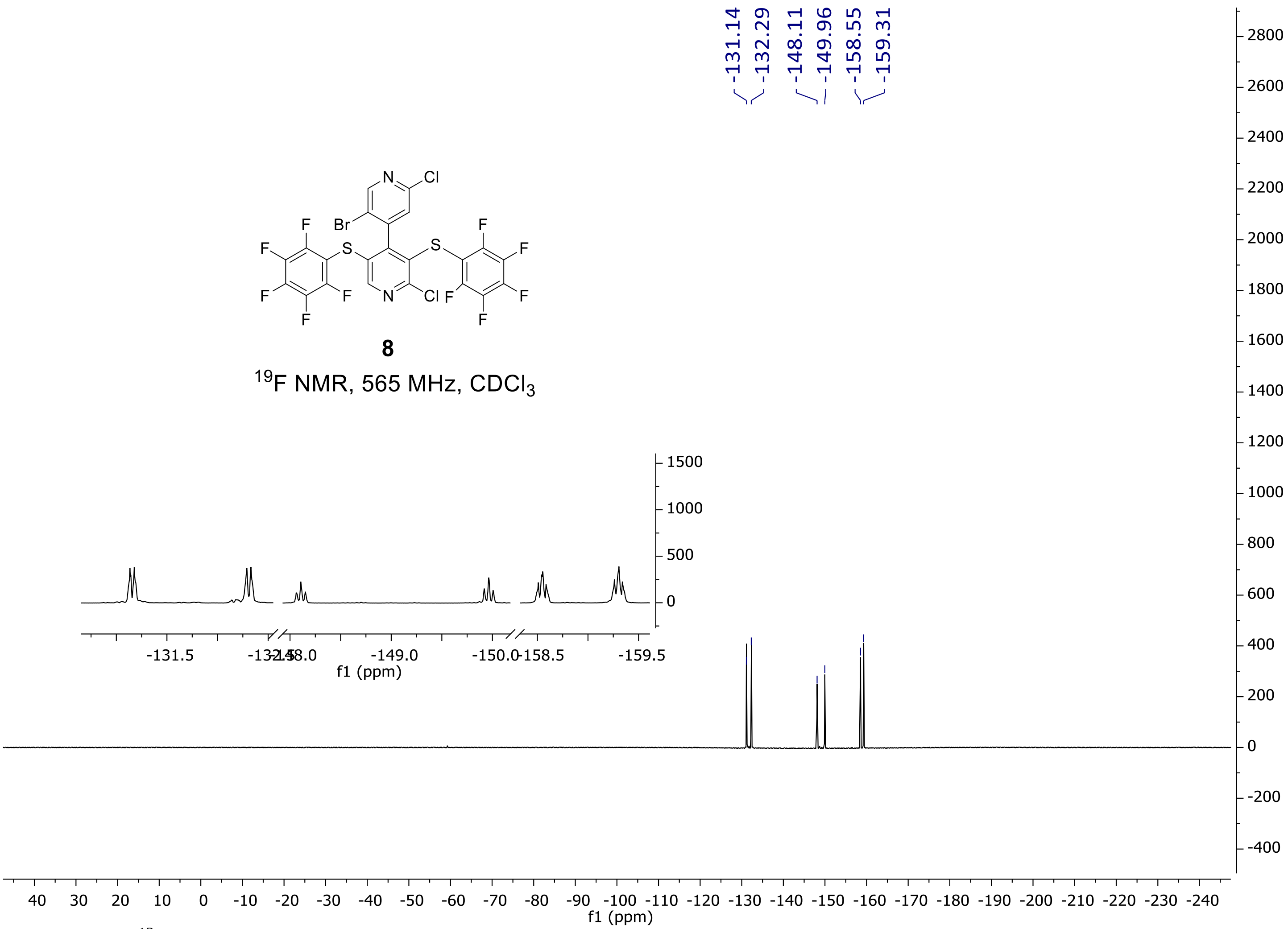

Figure S30. ^{13}C NMR spectrum of **8**

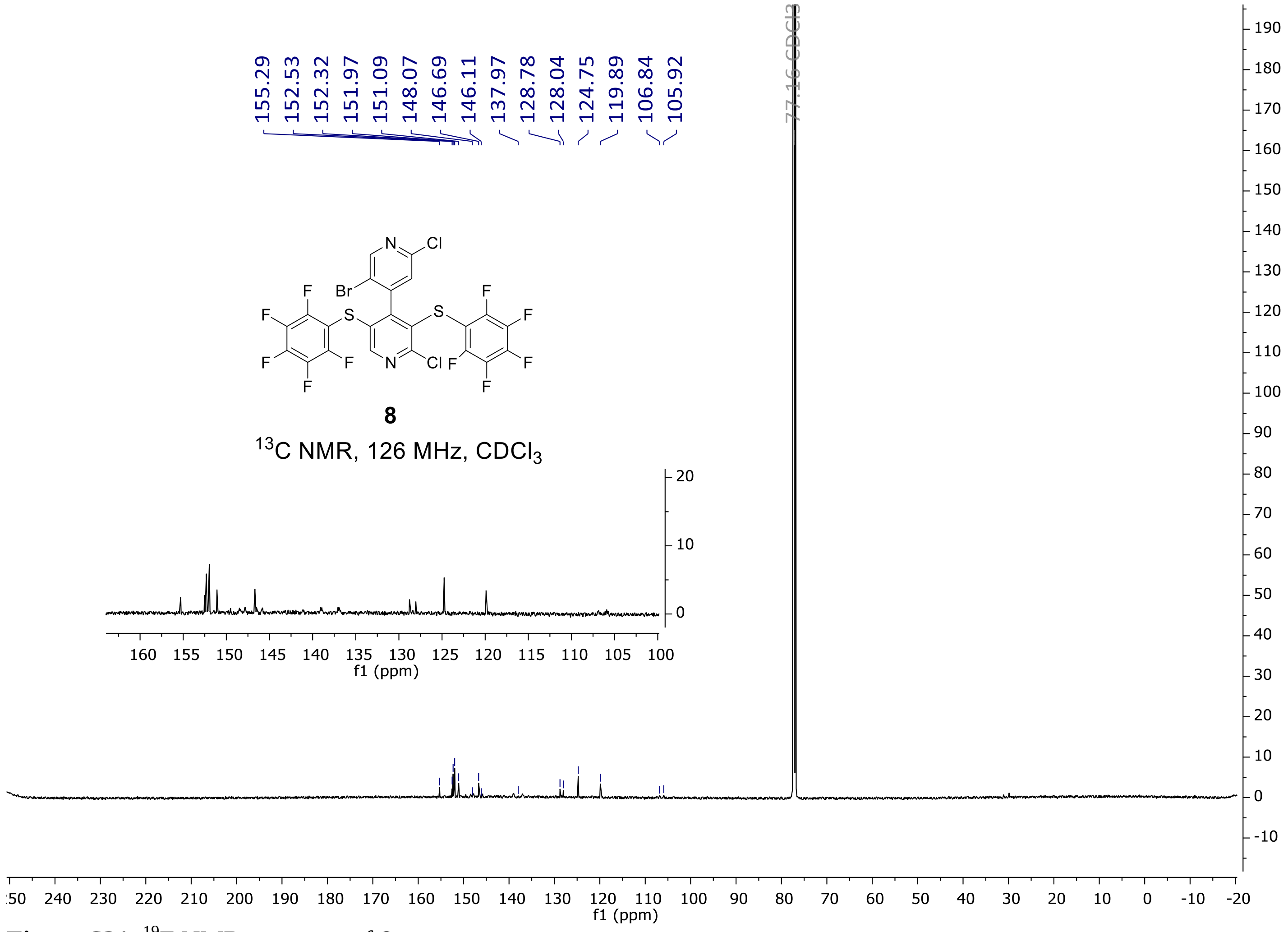

Figure S31. ^{19}F NMR spectrum of **8**

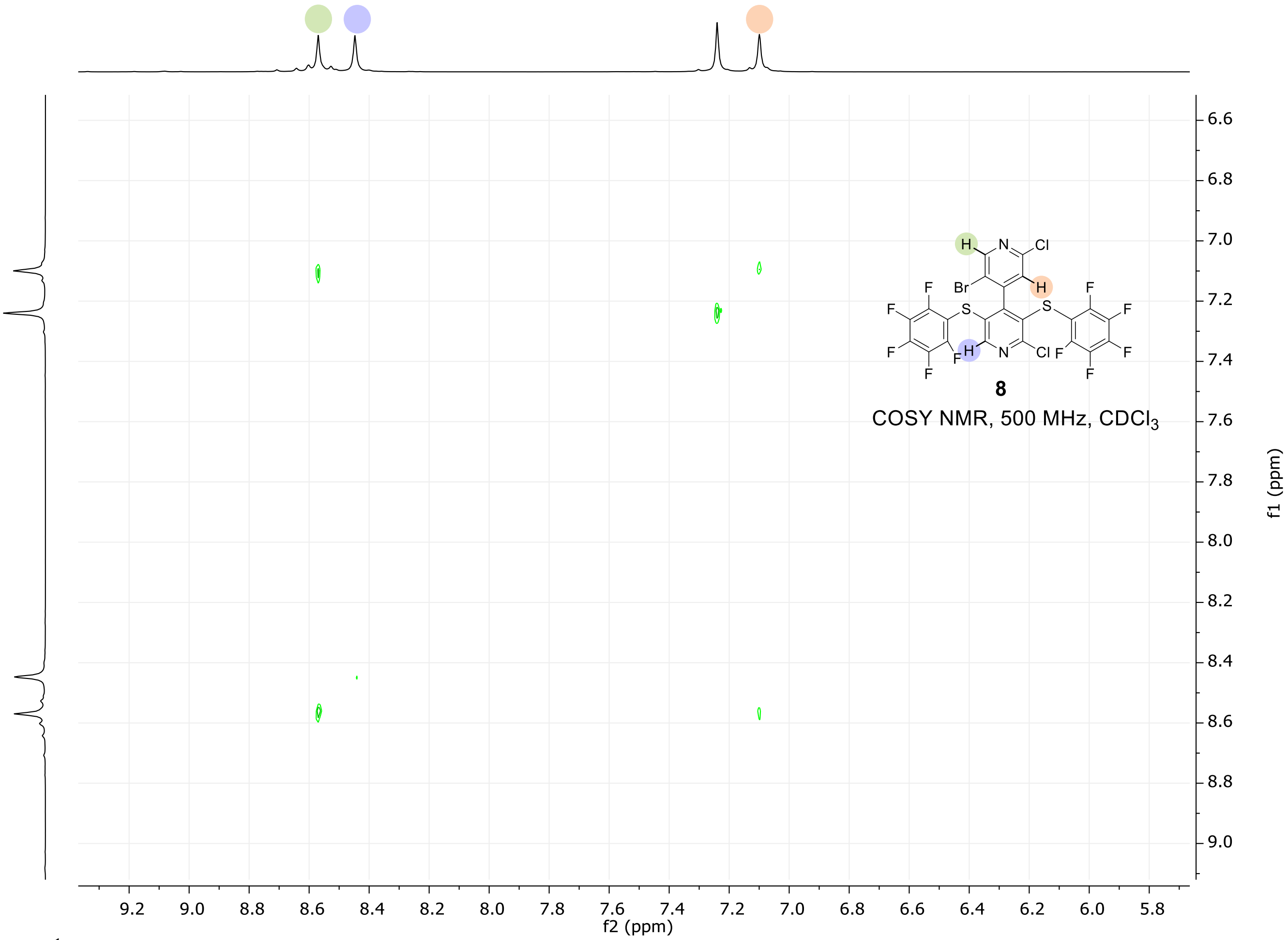

Figure S32. 2D ^{1}H NMR spectrum of **8** (NB: Hydrogens Green and Red are on the same pyridine)

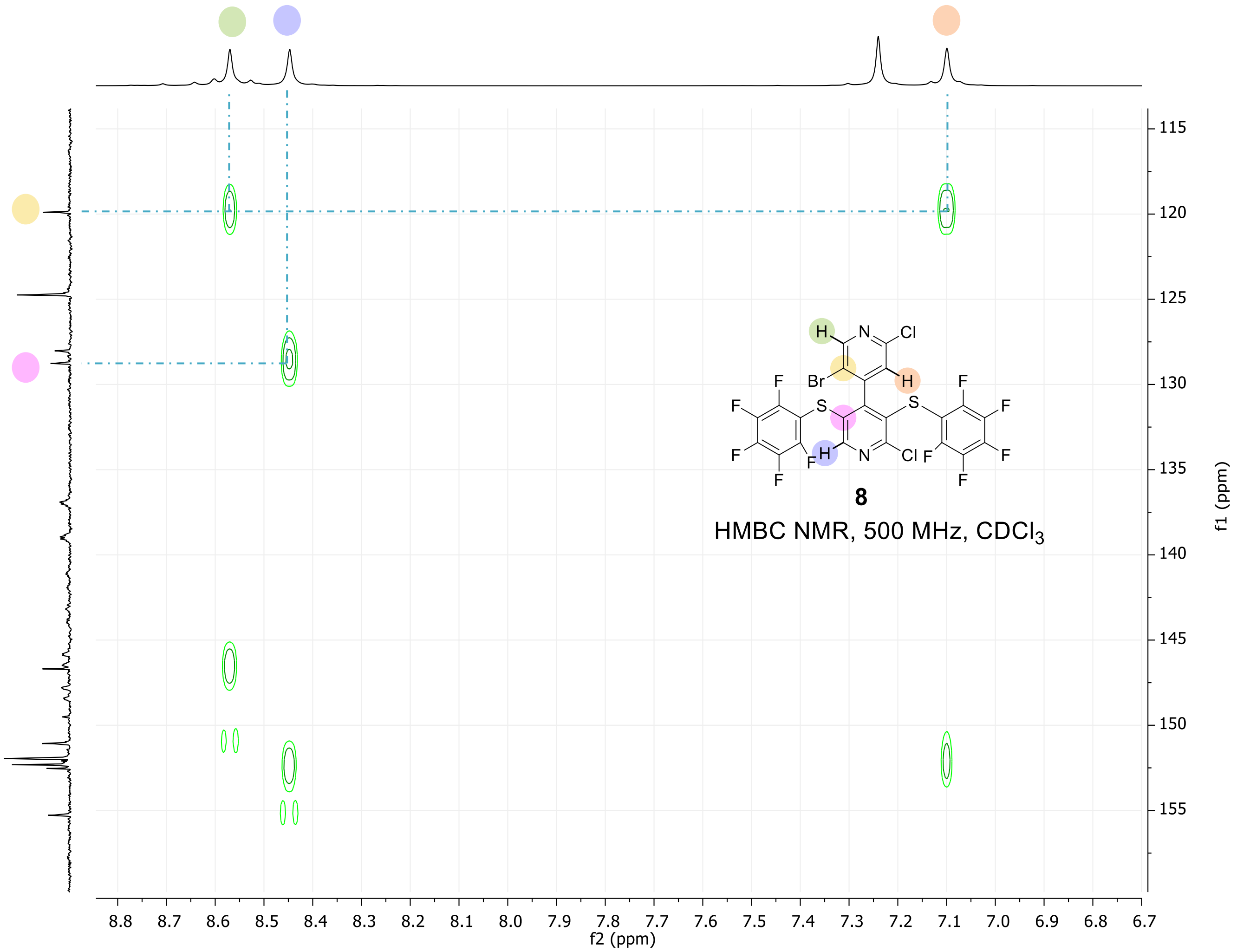

Figure S33. HMBC NMR spectrum of **8** (NB: Hydrogens Green and Red are in coupling with carbon Yellow which corresponds to C-Br (see Fig S25), Hydrogen Blue is in coupling with carbons Pink which correspond to C-S (see Fig S25)

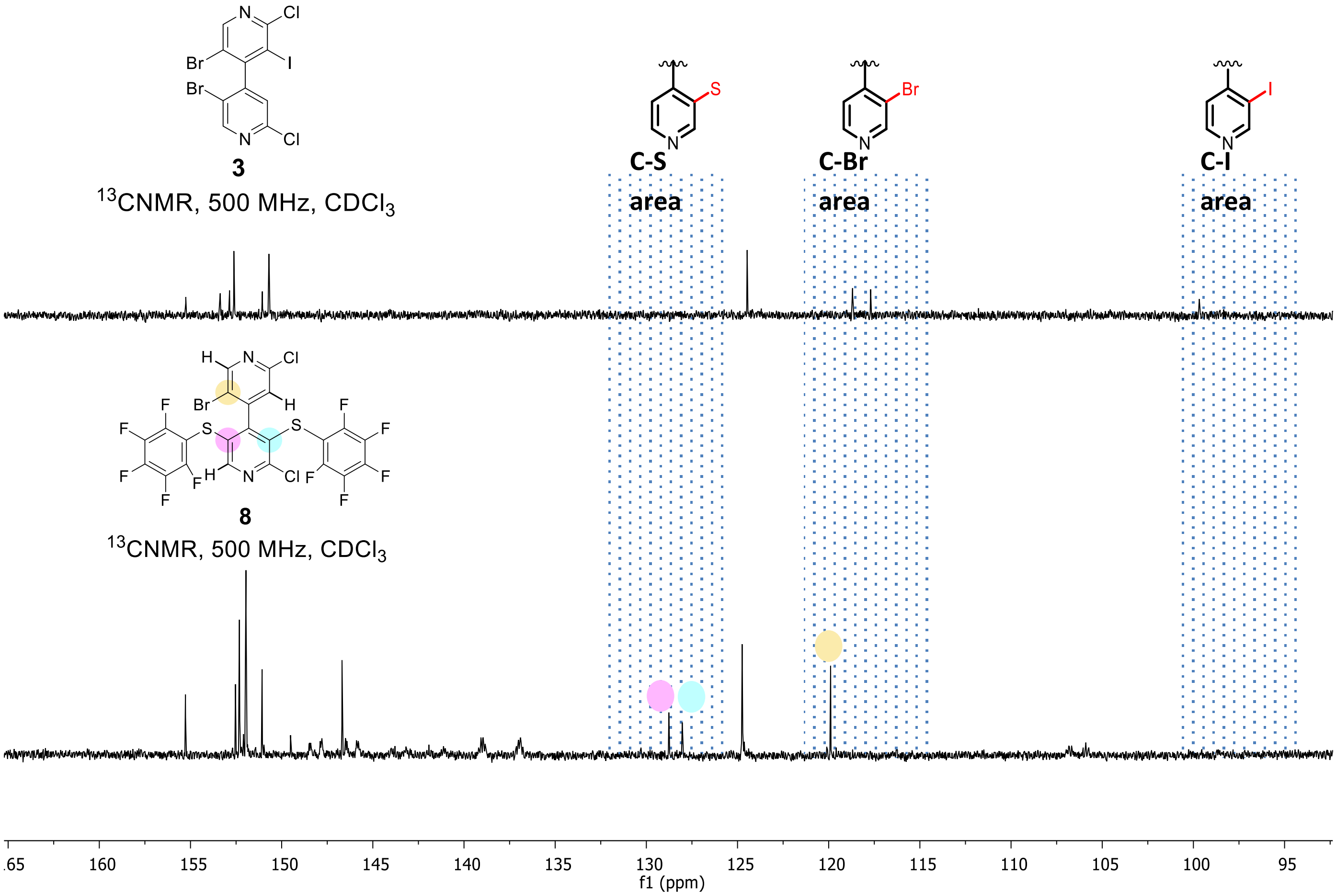

Figure S34. C-I, C-Br and C-S zones in ^{13}C NMR spectra of **3** and **8**
